# Supplementary material for: Polyketide synthase gene clusters in the ericoid mycorrhizal fungus Oidiodendron maius: genome-wide prediction, expression profiling, and functional characterization of knockout mutants
Source: Front Plant Sci. 2026 Sep 16;17:1924430. doi: 10.3389/fpls.2026.1924430 (PMC13623903; doi:10.3389/fpls.2026.1924430)
Supplement: Supplementary file 1 [file SupplementaryFile1.zip › Supplementary Materials/Supplementary Folder 1.zip!/Oidma1_AssemblyScaffolds_Repeatmasked/index.html]

scaffold\_1 - 59 clusters - antiSMASH results


antibiotics & Secondary Metabolite Analysis SHell  
Version 4.1.0

- Download all results
- Download XLS overview file
- Download EMBL summary file
- Download GenBank summary file

Select Gene Cluster:


- Overview
- 1
- 2
- 3
- 4
- 5
- 6
- 7
- 8
- 9
- 10
- 11
- 12
- 13
- 14
- 15
- 16
- 17
- 18
- 19
- 20
- 21
- 22
- 23
- 24
- 25
- 26
- 27
- 28
- 29
- 30
- 31
- 32
- 33
- 34
- 35
- 36
- 37
- 38
- 39
- 40
- 41
- 42
- 43
- 44
- 45
- 46
- 47
- 48
- 49
- 50
- 51
- 52
- 53
- 54
- 55
- 56
- 57
- 58
- 59

### Identified secondary metabolite clusters

| Cluster | Type | From | To | Most similar known cluster | MIBiG BGC-ID |
| --- | --- | --- | --- | --- | --- |
| The following clusters are from record scaffold\_1: | |  |  |  |  |
| Cluster 1 | Terpene | 294258 | 315546 | - | - |
| Cluster 2 | T1pks-Nrps | 379641 | 441215 | - | - |
| Cluster 3 | T1pks-Nrps | 458560 | 510849 | - | - |
| Cluster 4 | T1pks | 791591 | 838260 | Terrein\_biosynthetic\_gene\_cluster (27% of genes show similarity) | BGC0000161\_c1 |
| Cluster 5 | Other | 3276665 | 3320060 | - | - |
| Cluster 6 | Terpene | 4427280 | 4449975 | - | - |
| Cluster 7 | Other | 5458762 | 5502221 | - | - |
| Cluster 8 | Nrps | 6135134 | 6178888 | - | - |
| Cluster 9 | T1pks | 6227902 | 6276042 | - | - |
| The following clusters are from record scaffold\_2: | |  |  |  |  |
| Cluster 10 | Terpene | 548158 | 569334 | - | - |
| The following clusters are from record scaffold\_3: | |  |  |  |  |
| Cluster 11 | T1pks | 2078615 | 2124839 | - | - |
| Cluster 12 | Indole-T1pks | 2148048 | 2210787 | - | - |
| Cluster 13 | T1pks | 2628741 | 2720820 | Byssochlamic\_acid\_biosynthetic\_gene\_cluster (25% of genes show similarity) | BGC0001340\_c1 |
| The following clusters are from record scaffold\_4: | |  |  |  |  |
| Cluster 14 | T1pks | 1738950 | 1785709 | Beauvericin\_biosynthetic\_gene\_cluster (20% of genes show similarity) | BGC0000313\_c1 |
| The following clusters are from record scaffold\_5: | |  |  |  |  |
| Cluster 15 | Other | 398626 | 442199 | - | - |
| Cluster 16 | Other | 2091022 | 2134347 | - | - |
| Cluster 17 | Other | 2205493 | 2250549 | - | - |
| The following clusters are from record scaffold\_6: | |  |  |  |  |
| Cluster 18 | Terpene | 1916775 | 1938381 | - | - |
| Cluster 19 | T1pks | 2041350 | 2088122 | - | - |
| The following clusters are from record scaffold\_8: | |  |  |  |  |
| Cluster 20 | Nrps | 612777 | 667410 | - | - |
| Cluster 21 | T1pks | 1363091 | 1411375 | - | - |
| The following clusters are from record scaffold\_9: | |  |  |  |  |
| Cluster 22 | T1pks | 739406 | 787913 | - | - |
| Cluster 23 | T1pks | 937413 | 985702 | - | - |
| Cluster 24 | T1pks | 1072255 | 1134365 | - | - |
| Cluster 25 | T1pks | 1326212 | 1371648 | - | - |
| The following clusters are from record scaffold\_10: | |  |  |  |  |
| Cluster 26 | Other | 137993 | 181568 | - | - |
| The following clusters are from record scaffold\_11: | |  |  |  |  |
| Cluster 27 | T3pks-T1pks | 1184930 | 1238558 | - | - |
| The following clusters are from record scaffold\_12: | |  |  |  |  |
| Cluster 28 | T1pks | 373442 | 421357 | - | - |
| The following clusters are from record scaffold\_13: | |  |  |  |  |
| Cluster 29 | T1pks | 32112 | 79801 | - | - |
| Cluster 30 | T1pks | 246219 | 293290 | - | - |
| Cluster 31 | Nrps | 506748 | 554021 | - | - |
| Cluster 32 | T1pks-Nrps | 732574 | 784914 | Fusaridione\_A\_biosynthetic\_gene\_cluster (12% of genes show similarity) | BGC0000992\_c1 |
| Cluster 33 | T1pks | 895311 | 942376 | - | - |
| The following clusters are from record scaffold\_14: | |  |  |  |  |
| Cluster 34 | T1pks | 563359 | 610042 | - | - |
| Cluster 35 | Indole-T1pks | 719773 | 767883 | Neosartoricin\_biosynthetic\_gene\_cluster (60% of genes show similarity) | BGC0001144\_c1 |
| Cluster 36 | T1pks | 990688 | 1037285 | - | - |
| The following clusters are from record scaffold\_15: | |  |  |  |  |
| Cluster 37 | Other | 676610 | 719984 | - | - |
| The following clusters are from record scaffold\_16: | |  |  |  |  |
| Cluster 38 | Other | 221628 | 264778 | - | - |
| Cluster 39 | Other | 787561 | 830392 | - | - |
| The following clusters are from record scaffold\_17: | |  |  |  |  |
| Cluster 40 | T1pks | 121852 | 170035 | - | - |
| Cluster 41 | T1pks-Nrps | 250462 | 306229 | - | - |
| Cluster 42 | Nrps | 395683 | 440566 | Aspirochlorine\_biosynthetic\_gene\_cluster (18% of genes show similarity) | BGC0001123\_c1 |
| The following clusters are from record scaffold\_18: | |  |  |  |  |
| Cluster 43 | Siderophore | 589671 | 600870 | - | - |
| The following clusters are from record scaffold\_20: | |  |  |  |  |
| Cluster 44 | T1pks | 34834 | 81611 | - | - |
| Cluster 45 | T1pks | 498019 | 546308 | - | - |
| Cluster 46 | T1pks-Nrps | 795399 | 850123 | - | - |
| The following clusters are from record scaffold\_21: | |  |  |  |  |
| Cluster 47 | T1pks | 683224 | 738236 | Zearalenone\_biosynthetic\_gene\_cluster (18% of genes show similarity) | BGC0001057\_c1 |
| The following clusters are from record scaffold\_22: | |  |  |  |  |
| Cluster 48 | T1pks-Nrps | 8543 | 60967 | - | - |
| Cluster 49 | T1pks | 208990 | 292176 | Citrinin\_biosynthetic\_gene\_cluster (12% of genes show similarity) | BGC0001338\_c1 |
| The following clusters are from record scaffold\_23: | |  |  |  |  |
| Cluster 50 | T1pks-Nrps | 147253 | 213241 | Aflatrem\_biosynthetic\_gene\_cluster (11% of genes show similarity) | BGC0000629\_c1 |
| The following clusters are from record scaffold\_26: | |  |  |  |  |
| Cluster 51 | Indole-T1pks | 109140 | 176694 | Viridicatumtoxin\_biosynthetic\_gene\_cluster (40% of genes show similarity) | BGC0000168\_c1 |
| The following clusters are from record scaffold\_28: | |  |  |  |  |
| Cluster 52 | T1pks | 83384 | 138964 | - | - |
| Cluster 53 | T1pks-Nrps | 177158 | 232041 | - | - |
| The following clusters are from record scaffold\_33: | |  |  |  |  |
| Cluster 54 | T1pks | 59164 | 106713 | - | - |
| The following clusters are from record scaffold\_34: | |  |  |  |  |
| Cluster 55 | T1pks-Nrps | 25376 | 108672 | Trypacidin\_biosynthetic\_gene\_cluster (71% of genes show similarity) | BGC0001403\_c1 |
| The following clusters are from record scaffold\_35: | |  |  |  |  |
| Cluster 56 | T1pks-Nrps | 7178 | 101950 | - | - |
| Cluster 57 | Other | 110138 | 150436 | - | - |
| The following clusters are from record scaffold\_38: | |  |  |  |  |
| Cluster 58 | T1pks-Nrps | 25729 | 114735 | - | - |
| The following clusters are from record scaffold\_54: | |  |  |  |  |
| Cluster 59 | T1pks | 1 | 30982 | - | - |

### scaffold\_1 - Cluster 1 - Terpene

### Prediction details

jgi.p\_Oidma1\_107833
:   Cyclization pattern predicted: no prediction  
    Nearest neighbour: none found  
    Best known BLAST hit: (+)-Intermedeol Synthase (WP\_003955204, 27.4 %ID)

### Gene cluster description

Download cluster GenBank file

scaffold\_1 - Gene Cluster 1. Type = terpene. Location: 294258 - 315546 nt. Click on genes for more information.

Show pHMM detection rules used

terpene: (Terpene\_synth or Terpene\_synth\_C or phytoene\_synt or Lycopene\_cycl or terpene\_cyclase or NapT7 or fung\_ggpps or fung\_ggpps2 or trichodiene\_synth or TRI5)

#### Legend:

core biosynthetic genes

additional biosynthetic genes

transport-related genes

regulatory genes

other genes

cluster extent as predicted by CASSIS

### Homologous gene clusters

All hitsOidiodendron maius Zn unplaced genomic scaffold scaffold\_1, wh... (50% of genes show similarity)Penicillium camemberti str. FM013, whole genome shotgun sequen... (16% of genes show similarity)Aspergillus oryzae RIB40 DNA, SC023. (11% of genes show similarity)Aspergillus oryzae 3.042 Contig227.1, whole genome shotgun... (11% of genes show similarity)Aspergillus oryzae 100-8 Contig19.1, whole genome shotgun ... (11% of genes show similarity)Aspergillus flavus NRRL3357 scf\_1106286419142 genomic scaffold... (11% of genes show similarity)Aspergillus nomius NRRL 13137 contig00257, whole genome sh... (11% of genes show similarity)Talaromyces cellulolyticus DNA, scaffold: scf\_tce0\_042, strain... (11% of genes show similarity)Parastagonospora nodorum SN15 scaffold\_9, whole genome shotgun... (11% of genes show similarity)Download graphic

### scaffold\_1 - Cluster 2 - T1pks-nrps

### Predicted core structure

Rough prediction of core scaffold based on assumed PKS/NRPS colinearity; tailoring reactions not taken into account

### Prediction details

Monomers prediction:
:   N/A

### Database cross-links

Link to NORINE database query form  
Direct lookup in NORINE database in strict mode // relaxed mode

### Gene cluster description

Download cluster GenBank file

scaffold\_1 - Gene Cluster 2. Type = t1pks-nrps. Location: 379641 - 441215 nt. Click on genes for more information.

Show pHMM detection rules used

t1pks: ((PKS\_KS & PKS\_AT) or (ene\_KS & PKS\_AT) or (mod\_KS & PKS\_AT) or (hyb\_KS & PKS\_AT) or (itr\_KS & PKS\_AT) or (tra\_KS & PKS\_AT))  
nrps: ((Condensation & AMP-binding) or (Condensation & A-OX) or cluster(Condensation,AMP-binding))

#### Legend:

core biosynthetic genes

additional biosynthetic genes

transport-related genes

regulatory genes

other genes

cluster extent as predicted by CASSIS

### Detailed annotation

### Homologous gene clusters

All hitsOidiodendron maius Zn unplaced genomic scaffold scaffold\_1, wh... (100% of genes show similarity)Aspergillus terreus NIH2624 scaffold\_8 genomic scaffold, whole... (21% of genes show similarity)Talaromyces cellulolyticus DNA, scaffold: scf\_tce0\_013, strain... (17% of genes show similarity)Arthroderma gypseum CBS 118893 supercont1.4 genomic scaffold, ... (21% of genes show similarity)Pseudogymnoascus sp. VKM F-103 contig\_540, whole genome sh... (13% of genes show similarity)Pseudogymnoascus sp. VKM F-4519 (FW-2642) contig\_1966, who... (13% of genes show similarity)Pestalotiopsis fici W106-1 unplaced genomic scaffold PFICI\_Scf... (13% of genes show similarity)Fusarium fujikuroi fusarubin biosynthesis gene cluster, strain... (13% of genes show similarity)Fusarium fujikuroi IMI 58289 draft genome, chromosome FFUJ\_chr... (13% of genes show similarity)Bipolaris victoriae FI3 unplaced genomic scaffold scaffold\_24... (13% of genes show similarity)Download graphic

### scaffold\_1 - Cluster 3 - T1pks-nrps

### Predicted core structure

Rough prediction of core scaffold based on assumed PKS/NRPS colinearity; tailoring reactions not taken into account

### Prediction details

Monomers prediction:
:   N/A

### Database cross-links

Link to NORINE database query form  
Direct lookup in NORINE database in strict mode // relaxed mode

### Gene cluster description

Download cluster GenBank file

scaffold\_1 - Gene Cluster 3. Type = t1pks-nrps. Location: 458560 - 510849 nt. Click on genes for more information.

Show pHMM detection rules used

t1pks: ((PKS\_KS & PKS\_AT) or (ene\_KS & PKS\_AT) or (mod\_KS & PKS\_AT) or (hyb\_KS & PKS\_AT) or (itr\_KS & PKS\_AT) or (tra\_KS & PKS\_AT))  
nrps: ((Condensation & AMP-binding) or (Condensation & A-OX) or cluster(Condensation,AMP-binding))

#### Legend:

core biosynthetic genes

additional biosynthetic genes

transport-related genes

regulatory genes

other genes

cluster extent as predicted by CASSIS

### Detailed annotation

### Homologous gene clusters

All hitsOidiodendron maius Zn unplaced genomic scaffold scaffold\_1, wh... (100% of genes show similarity)Penicillium italicum strain PHI-1 contig2416, whole genome... (37% of genes show similarity)Podospora anserina S mat+ genomic DNA chromosome 5, superconti... (31% of genes show similarity)Podospora anserina genomic DNA, chromosome 5. (25% of genes show similarity)Talaromyces islandicus genome assembly PIS, scaffold WF-3... (31% of genes show similarity)Trichoderma harzianum strain T6776 Scaffolds0020.1, whole ... (25% of genes show similarity)Bipolaris victoriae FI3 unplaced genomic scaffold scaffold\_71,... (25% of genes show similarity)Stachybotrys chartarum IBT 7711 unplaced genomic scaffold scaf... (31% of genes show similarity)Stachybotrys chartarum IBT 40293 unplaced genomic scaffold sca... (25% of genes show similarity)Oidiodendron maius Zn unplaced genomic scaffold scaffold\_21, ... (25% of genes show similarity)Download graphic

### scaffold\_1 - Cluster 4 - T1pks

### Predicted core structure

Rough prediction of core scaffold based on assumed PKS/NRPS colinearity; tailoring reactions not taken into account

### Prediction details

Monomers prediction:
:   N/A

### Gene cluster description

Download cluster GenBank file

scaffold\_1 - Gene Cluster 4. Type = t1pks. Location: 791591 - 838260 nt. Click on genes for more information.

Show pHMM detection rules used

t1pks: ((PKS\_KS & PKS\_AT) or (ene\_KS & PKS\_AT) or (mod\_KS & PKS\_AT) or (hyb\_KS & PKS\_AT) or (itr\_KS & PKS\_AT) or (tra\_KS & PKS\_AT))

#### Legend:

core biosynthetic genes

additional biosynthetic genes

transport-related genes

regulatory genes

other genes

cluster extent as predicted by CASSIS

### Detailed annotation

### Homologous gene clusters

All hitsOidiodendron maius Zn unplaced genomic scaffold scaffold\_1, wh... (100% of genes show similarity)Aspergillus terreus NIH2624 scaffold\_1 genomic scaffold, whole... (38% of genes show similarity)Aspergillus lentulus DNA, contig: Alt01, strain: IFM 5470... (38% of genes show similarity)Nostoc sp. ATCC 53789 cryptophycin biosynthetic gene cluster, ... (23% of genes show similarity)Glomerella graminicola M1.001 genomic scaffold supercont1.42, ... (46% of genes show similarity)Aspergillus ustus strain 3.3904 AUSC\_33, whole genome shot... (30% of genes show similarity)Trichophyton interdigitale MR816 cont1.257, whole genome s... (23% of genes show similarity)Trichophyton interdigitale H6 unplaced genomic scaffold superc... (23% of genes show similarity)Trichophyton rubrum strain IGIB-SBL-CI1 Contig006, whole g... (23% of genes show similarity)Arthroderma gypseum CBS 118893 supercont1.10 genomic scaffold... (23% of genes show similarity)Download graphic

### Homologous known gene clusters

All hitsTerrein biosynthetic gene cluster (27% of genes show similarity)Download graphic

### scaffold\_1 - Cluster 5 - Other

### Predicted core structure

Rough prediction of core scaffold based on assumed PKS/NRPS colinearity; tailoring reactions not taken into account

### Prediction details

Monomers prediction:
:   N/A

### Gene cluster description

Download cluster GenBank file

scaffold\_1 - Gene Cluster 5. Type = other. Location: 3276665 - 3320060 nt. Click on genes for more information.

Show pHMM detection rules used

other: ((PP-binding & AMP-binding) or (PP-binding & A-OX) or (NAD\_binding\_4 & A-OX) or (NAD\_binding\_4 & AMP-binding) or LmbU or Neocarzinostat or cycdipepsynth or fom1 or bcpB or frbD or mitE or vlmB or prnB or CaiA or bacilysin or orf2\_PTase)

#### Legend:

core biosynthetic genes

additional biosynthetic genes

transport-related genes

regulatory genes

other genes

cluster extent as predicted by CASSIS

### Detailed annotation

### Homologous gene clusters

All hitsOidiodendron maius Zn unplaced genomic scaffold scaffold\_1, wh... (100% of genes show similarity)Torrubiella hemipterigena genome assembly Allpaths-LG, sca... (23% of genes show similarity)Fonsecaea multimorphosa CBS 102226 unplaced genomic scaffold s... (15% of genes show similarity)Rhinocladiella mackenziei CBS 650.93 unplaced genomic scaffold... (15% of genes show similarity)Exophiala aquamarina CBS 119918 aczIA-supercont1.5, whole ... (15% of genes show similarity)Exophiala spinifera strain CBS 89968 unplaced genomic scaffold... (15% of genes show similarity)Streptomyces turgidiscabies Car8 gcontig\_1113219320867, wh... (15% of genes show similarity)Parastagonospora nodorum SN15 scaffold\_24, whole genome shotgu... (15% of genes show similarity)Stachybotrys chlorohalonata IBT 40285 unplaced genomic scaffol... (15% of genes show similarity)Stachybotrys chartarum IBT 40293 unplaced genomic scaffold sc... (15% of genes show similarity)Download graphic

### scaffold\_1 - Cluster 6 - Terpene

### Prediction details

jgi.p\_Oidma1\_108948
:   Cyclization pattern predicted: no prediction  
    Nearest neighbour: none found  
    Best known BLAST hit: 7-epi-a-Eudesmol Synthase (WP\_029384838, 37.2 %ID)

### Gene cluster description

Download cluster GenBank file

scaffold\_1 - Gene Cluster 6. Type = terpene. Location: 4427280 - 4449975 nt. Click on genes for more information.

Show pHMM detection rules used

terpene: (Terpene\_synth or Terpene\_synth\_C or phytoene\_synt or Lycopene\_cycl or terpene\_cyclase or NapT7 or fung\_ggpps or fung\_ggpps2 or trichodiene\_synth or TRI5)

#### Legend:

core biosynthetic genes

additional biosynthetic genes

transport-related genes

regulatory genes

other genes

cluster extent as predicted by CASSIS

### Homologous gene clusters

All hitsOidiodendron maius Zn unplaced genomic scaffold scaffold\_1, wh... (64% of genes show similarity)Coccidioides posadasii RMSCC 3488 supercont1.5 genomic scaffol... (21% of genes show similarity)Coccidioides immitis RS genomic scaffold supercont3.5, whole g... (21% of genes show similarity)Coccidioides posadasii str. Silveira unplaced genomic scaffold... (21% of genes show similarity)Aspergillus clavatus NRRL 1 1099423829791 genomic scaffold, wh... (21% of genes show similarity)Bipolaris maydis ATCC 48331 unplaced genomic scaffold COCC4sca... (21% of genes show similarity)Cochliobolus heterostrophus C5 unplaced genomic scaffold COCHE... (21% of genes show similarity)Bipolaris oryzae ATCC 44560 unplaced genomic scaffold scaffold... (21% of genes show similarity)Beauveria bassiana ARSEF 2860 unplaced genomic scaffold BBA\_S0... (21% of genes show similarity)Beauveria bassiana D1-5 contig00822, whole genome shotgun... (21% of genes show similarity)Download graphic

### scaffold\_1 - Cluster 7 - Other

### Predicted core structure

Rough prediction of core scaffold based on assumed PKS/NRPS colinearity; tailoring reactions not taken into account

### Prediction details

Monomers prediction:
:   N/A

### Gene cluster description

Download cluster GenBank file

scaffold\_1 - Gene Cluster 7. Type = other. Location: 5458762 - 5502221 nt. Click on genes for more information.

Show pHMM detection rules used

other: ((PP-binding & AMP-binding) or (PP-binding & A-OX) or (NAD\_binding\_4 & A-OX) or (NAD\_binding\_4 & AMP-binding) or LmbU or Neocarzinostat or cycdipepsynth or fom1 or bcpB or frbD or mitE or vlmB or prnB or CaiA or bacilysin or orf2\_PTase)

#### Legend:

core biosynthetic genes

additional biosynthetic genes

transport-related genes

regulatory genes

other genes

cluster extent as predicted by CASSIS

### Detailed annotation

### Homologous gene clusters

All hitsOidiodendron maius Zn unplaced genomic scaffold scaffold\_1, wh... (100% of genes show similarity)Aspergillus parasiticus SU-1 396, whole genome shotgun seq... (15% of genes show similarity)Trichosporon asahii var. asahii CBS 2479 contig0180, whole... (10% of genes show similarity)Trichosporon asahii var. asahii CBS 8904 contig0402, whole... (10% of genes show similarity)Magnaporthe oryzae Y34 unplaced genomic scaffold Y34\_scaffold0... (26% of genes show similarity)Magnaporthe oryzae 70-15 chromosome 3, whole genome shotgun se... (26% of genes show similarity)Gaeumannomyces graminis var. tritici R3-111a-1 unplaced genomi... (26% of genes show similarity)Penicillium brasilianum genome assembly Pbras\_Allpaths-LG,... (15% of genes show similarity)Talaromyces islandicus genome assembly PIS, scaffold WF-38... (10% of genes show similarity)Magnaporthe poae ATCC 64411 unplaced genomic scaffold superco... (21% of genes show similarity)Download graphic

### scaffold\_1 - Cluster 8 - Nrps

### Predicted core structure

Rough prediction of core scaffold based on assumed PKS/NRPS colinearity; tailoring reactions not taken into account

### Prediction details

Monomers prediction:
:   N/A

### Database cross-links

Link to NORINE database query form  
Direct lookup in NORINE database in strict mode // relaxed mode

### Gene cluster description

Download cluster GenBank file

scaffold\_1 - Gene Cluster 8. Type = nrps. Location: 6135134 - 6178888 nt. Click on genes for more information.

Show pHMM detection rules used

nrps: ((Condensation & AMP-binding) or (Condensation & A-OX) or cluster(Condensation,AMP-binding))

#### Legend:

core biosynthetic genes

additional biosynthetic genes

transport-related genes

regulatory genes

other genes

cluster extent as predicted by CASSIS

### Detailed annotation

### Homologous gene clusters

All hitsOidiodendron maius Zn unplaced genomic scaffold scaffold\_1, wh... (100% of genes show similarity)Dothistroma septosporum NZE10 unplaced genomic scaffold DOTSEs... (11% of genes show similarity)Metarhizium album ARSEF 1941 scaffold\_37, whole genome sho... (11% of genes show similarity)Penicillium camemberti str. FM013, whole genome shotgun sequen... (11% of genes show similarity)Penicillium solitum strain RS1 scf7180000043680, whole gen... (11% of genes show similarity)Penicillium nordicum strain DAOMC 185683 scaffold\_86, whol... (11% of genes show similarity)Penicillium chrysogenum Wisconsin 54-1255 complete genome, con... (11% of genes show similarity)Aspergillus niger ATCC 1015, whole genome shotgun sequence. (16% of genes show similarity)Aspergillus niger contig An18c0020, genomic contig. (16% of genes show similarity)Aspergillus kawachii IFO 4308 DNA, contig: scaffold00042, who... (16% of genes show similarity)Download graphic

### scaffold\_1 - Cluster 9 - T1pks

### Predicted core structure

Rough prediction of core scaffold based on assumed PKS/NRPS colinearity; tailoring reactions not taken into account

### Prediction details

Monomers prediction:
:   N/A

### Gene cluster description

Download cluster GenBank file

scaffold\_1 - Gene Cluster 9. Type = t1pks. Location: 6227902 - 6276042 nt. Click on genes for more information.

Show pHMM detection rules used

t1pks: ((PKS\_KS & PKS\_AT) or (ene\_KS & PKS\_AT) or (mod\_KS & PKS\_AT) or (hyb\_KS & PKS\_AT) or (itr\_KS & PKS\_AT) or (tra\_KS & PKS\_AT))

#### Legend:

core biosynthetic genes

additional biosynthetic genes

transport-related genes

regulatory genes

other genes

cluster extent as predicted by CASSIS

### Detailed annotation

### Homologous gene clusters

All hitsOidiodendron maius Zn unplaced genomic scaffold scaffold\_1, wh... (100% of genes show similarity)Aspergillus kawachii IFO 4308 DNA, contig: scaffold00008, whol... (26% of genes show similarity)Colletotrichum gloeosporioides Nara gc5 unplaced genomic scaff... (20% of genes show similarity)Colletotrichum fioriniae PJ7 Scaffold\_495.1, whole genome ... (20% of genes show similarity)Aspergillus niger contig An11c0230, genomic contig. (20% of genes show similarity)Aspergillus niger contig An12c0070, genomic contig. (20% of genes show similarity)Talaromyces marneffei PM1 scf033, whole genome shotgun seq... (13% of genes show similarity)Neosartorya udagawae DNA, contig:Aud0186, strain: IFM 4697... (13% of genes show similarity)Gaeumannomyces graminis var. tritici R3-111a-1 unplaced genomi... (13% of genes show similarity)Chaetomium globosum CBS 148.51 scaffold\_2 genomic scaffold, w... (13% of genes show similarity)Download graphic

### scaffold\_2 - Cluster 10 - Terpene

### Gene cluster description

Download cluster GenBank file

scaffold\_2 - Gene Cluster 10. Type = terpene. Location: 548158 - 569334 nt. Click on genes for more information.

Show pHMM detection rules used

terpene: (Terpene\_synth or Terpene\_synth\_C or phytoene\_synt or Lycopene\_cycl or terpene\_cyclase or NapT7 or fung\_ggpps or fung\_ggpps2 or trichodiene\_synth or TRI5)

#### Legend:

core biosynthetic genes

additional biosynthetic genes

transport-related genes

regulatory genes

other genes

cluster extent as predicted by CASSIS

### Homologous gene clusters

All hitsOidiodendron maius Zn unplaced genomic scaffold scaffold\_2, wh... (60% of genes show similarity)Botryotinia fuckeliana BcDW1 unplaced genomic scaffold Scaffol... (26% of genes show similarity)Togninia minima UCRPA7 unplaced genomic scaffold PA7\_03\_scaffo... (20% of genes show similarity)Glarea lozoyensis ATCC 20868 chromosome Unknown GLAREA2, whole... (20% of genes show similarity)Pseudogymnoascus sp. VKM F-4520 (FW-2644) contig\_2898, who... (20% of genes show similarity)Pseudogymnoascus sp. VKM F-103 contig\_583, whole genome sh... (20% of genes show similarity)Neonectria ditissima strain R09/05 contig\_15, whole genome... (20% of genes show similarity)Tolypocladium ophioglossoides CBS 100239 scaffold\_7, whole... (20% of genes show similarity)Metarhizium acridum CQMa 102 unplaced genomic scaffold Scf\_013... (20% of genes show similarity)Metarhizium robertsii strain ARSEF 2575 contig005, whole ... (20% of genes show similarity)Download graphic

### scaffold\_3 - Cluster 11 - T1pks

### Predicted core structure

Rough prediction of core scaffold based on assumed PKS/NRPS colinearity; tailoring reactions not taken into account

### Prediction details

Monomers prediction:
:   N/A

### Gene cluster description

Download cluster GenBank file

scaffold\_3 - Gene Cluster 11. Type = t1pks. Location: 2078615 - 2124839 nt. Click on genes for more information.

Show pHMM detection rules used

t1pks: ((PKS\_KS & PKS\_AT) or (ene\_KS & PKS\_AT) or (mod\_KS & PKS\_AT) or (hyb\_KS & PKS\_AT) or (itr\_KS & PKS\_AT) or (tra\_KS & PKS\_AT))

#### Legend:

core biosynthetic genes

additional biosynthetic genes

transport-related genes

regulatory genes

other genes

cluster extent as predicted by CASSIS

### Detailed annotation

### Homologous gene clusters

All hitsOidiodendron maius Zn unplaced genomic scaffold scaffold\_3, wh... (100% of genes show similarity)Glarea lozoyensis ATCC 20868 chromosome Unknown GLAREA14, whol... (28% of genes show similarity)Pseudogymnoascus sp. VKM F-4516 (FW-969) contig\_50, whole ... (28% of genes show similarity)Cordyceps militaris CM01 unplaced genomic scaffold CCM\_S00003,... (21% of genes show similarity)Pseudogymnoascus sp. VKM F-3808 contig\_1320, whole genome ... (21% of genes show similarity)Pseudogymnoascus sp. VKM F-4514 (FW-929) contig\_65, whole ... (21% of genes show similarity)Diaporthe ampelina DA\_912\_scaffold\_v01\_90, whole genome sh... (21% of genes show similarity)Hirsutella minnesotensis 3608 unplaced genomic scaffold MNSD\_S... (35% of genes show similarity)Neosartorya fischeri NRRL 181 1099437636245 genomic scaffold, ... (28% of genes show similarity)Geomyces destructans 20631-21 unplaced genomic scaffold super... (28% of genes show similarity)Download graphic

### scaffold\_3 - Cluster 12 - Indole-t1pks

### Predicted core structure

Rough prediction of core scaffold based on assumed PKS/NRPS colinearity; tailoring reactions not taken into account

### Prediction details

Monomers prediction:
:   N/A

### Gene cluster description

Download cluster GenBank file

scaffold\_3 - Gene Cluster 12. Type = indole-t1pks. Location: 2148048 - 2210787 nt. Click on genes for more information.

Show pHMM detection rules used

indole: (indsynth or dmat or indole\_PTase)  
t1pks: ((PKS\_KS & PKS\_AT) or (ene\_KS & PKS\_AT) or (mod\_KS & PKS\_AT) or (hyb\_KS & PKS\_AT) or (itr\_KS & PKS\_AT) or (tra\_KS & PKS\_AT))

#### Legend:

core biosynthetic genes

additional biosynthetic genes

transport-related genes

regulatory genes

other genes

cluster extent as predicted by CASSIS

### Detailed annotation

### Homologous gene clusters

All hitsOidiodendron maius Zn unplaced genomic scaffold scaffold\_3, wh... (100% of genes show similarity)Stachybotrys chartarum IBT 7711 unplaced genomic scaffold scaf... (50% of genes show similarity)Stachybotrys chartarum IBT 40293 unplaced genomic scaffold sca... (50% of genes show similarity)Stachybotrys chartarum IBT 40288 unplaced genomic scaffold sca... (40% of genes show similarity)Setosphaeria turcica Et28A unplaced genomic scaffold SETTUscaf... (45% of genes show similarity)Penicillium camemberti str. FM013, whole genome shotgun sequen... (36% of genes show similarity)Penicillium digitatum Pd1 contig158, whole genome shotgun ... (31% of genes show similarity)Penicillium digitatum PHI26 contig102, whole genome shotgu... (31% of genes show similarity)Glarea lozoyensis ATCC 20868 chromosome Unknown GLAREA5, whole... (31% of genes show similarity)Thielavia terrestris NRRL 8126 chromosome 3, complete sequence. (36% of genes show similarity)Download graphic

### scaffold\_3 - Cluster 13 - T1pks

### Predicted core structure

Rough prediction of core scaffold based on assumed PKS/NRPS colinearity; tailoring reactions not taken into account

### Prediction details

Monomers prediction:
:   N/A

### Gene cluster description

Download cluster GenBank file

scaffold\_3 - Gene Cluster 13. Type = t1pks. Location: 2628741 - 2720820 nt. Click on genes for more information.

Show pHMM detection rules used

t1pks: ((PKS\_KS & PKS\_AT) or (ene\_KS & PKS\_AT) or (mod\_KS & PKS\_AT) or (hyb\_KS & PKS\_AT) or (itr\_KS & PKS\_AT) or (tra\_KS & PKS\_AT))

#### Legend:

core biosynthetic genes

additional biosynthetic genes

transport-related genes

regulatory genes

other genes

cluster extent as predicted by CASSIS

### Detailed annotation

### Homologous gene clusters

All hitsOidiodendron maius Zn unplaced genomic scaffold scaffold\_3, wh... (100% of genes show similarity)Talaromyces stipitatus ATCC 10500 scf\_1105507295555 genomic sc... (20% of genes show similarity)Bipolaris zeicola 26-R-13 unplaced genomic scaffold scaffold\_1... (24% of genes show similarity)Bipolaris victoriae FI3 unplaced genomic scaffold scaffold\_82,... (24% of genes show similarity)Bipolaris maydis ATCC 48331 unplaced genomic scaffold COCC4sca... (24% of genes show similarity)Cochliobolus heterostrophus C5 unplaced genomic scaffold COCHE... (24% of genes show similarity)Talaromyces stipitatus ATCC 10500 scf\_1105507295515 genomic sc... (20% of genes show similarity)Aureobasidium subglaciale EXF-2481 unplaced genomic scaffold s... (20% of genes show similarity)Neosartorya udagawae DNA, contig:Aud0116, strain: IFM 4697... (10% of genes show similarity)Exophiala xenobiotica strain CBS 118157 unplaced genomic scaf... (10% of genes show similarity)Download graphic

### Homologous known gene clusters

All hitsByssochlamic acid biosynthetic gene cluster (25% of genes show similarity)Byssochlamic acid / agnestadrides biosynthetic gene cluster (25% of genes show similarity)Download graphic

### scaffold\_4 - Cluster 14 - T1pks

### Predicted core structure

Rough prediction of core scaffold based on assumed PKS/NRPS colinearity; tailoring reactions not taken into account

### Prediction details

Monomers prediction:
:   N/A

### Gene cluster description

Download cluster GenBank file

scaffold\_4 - Gene Cluster 14. Type = t1pks. Location: 1738950 - 1785709 nt. Click on genes for more information.

Show pHMM detection rules used

t1pks: ((PKS\_KS & PKS\_AT) or (ene\_KS & PKS\_AT) or (mod\_KS & PKS\_AT) or (hyb\_KS & PKS\_AT) or (itr\_KS & PKS\_AT) or (tra\_KS & PKS\_AT))

#### Legend:

core biosynthetic genes

additional biosynthetic genes

transport-related genes

regulatory genes

other genes

cluster extent as predicted by CASSIS

### Detailed annotation

### Homologous gene clusters

All hitsOidiodendron maius Zn unplaced genomic scaffold scaffold\_4, wh... (100% of genes show similarity)Geomyces destructans 20631-21 unplaced genomic scaffold superc... (26% of genes show similarity)Pseudogymnoascus sp. VKM F-4281 (FW-2241) contig\_1900, who... (26% of genes show similarity)Pseudogymnoascus sp. VKM F-103 contig\_765, whole genome sh... (26% of genes show similarity)Pseudogymnoascus sp. VKM F-4519 (FW-2642) contig\_433, whol... (21% of genes show similarity)Pestalotiopsis fici W106-1 unplaced genomic scaffold PFICI\_Scf... (21% of genes show similarity)Ceratocystis platani strain CFO contig\_00013, whole genome... (21% of genes show similarity)Pseudogymnoascus sp. VKM F-4515 (FW-2607) contig\_1673, who... (21% of genes show similarity)Pseudogymnoascus sp. VKM F-4516 (FW-969) contig\_50, whole ... (21% of genes show similarity)Pseudogymnoascus sp. VKM F-4514 (FW-929) contig\_65, whole... (21% of genes show similarity)Download graphic

### Homologous known gene clusters

All hitsBeauvericin biosynthetic gene cluster (20% of genes show similarity)Download graphic

### scaffold\_5 - Cluster 15 - Other

### Predicted core structure

Rough prediction of core scaffold based on assumed PKS/NRPS colinearity; tailoring reactions not taken into account

### Prediction details

Monomers prediction:
:   N/A

### Gene cluster description

Download cluster GenBank file

scaffold\_5 - Gene Cluster 15. Type = other. Location: 398626 - 442199 nt. Click on genes for more information.

Show pHMM detection rules used

other: ((PP-binding & AMP-binding) or (PP-binding & A-OX) or (NAD\_binding\_4 & A-OX) or (NAD\_binding\_4 & AMP-binding) or LmbU or Neocarzinostat or cycdipepsynth or fom1 or bcpB or frbD or mitE or vlmB or prnB or CaiA or bacilysin or orf2\_PTase)

#### Legend:

core biosynthetic genes

additional biosynthetic genes

transport-related genes

regulatory genes

other genes

cluster extent as predicted by CASSIS

### Detailed annotation

### Homologous gene clusters

All hitsOidiodendron maius Zn unplaced genomic scaffold scaffold\_5, wh... (100% of genes show similarity)Marssonina brunnea f. sp. 'multigermtubi' MB\_m1 unplaced genom... (38% of genes show similarity)Botryotinia fuckeliana BcDW1 unplaced genomic scaffold Scaffol... (33% of genes show similarity)Botryotinia fuckeliana T4 SuperContig\_219\_1 genomic supercontig. (33% of genes show similarity)Sclerotinia sclerotiorum 1980 scaffold\_11 genomic scaffold, wh... (38% of genes show similarity)Sclerotinia borealis F-4157 contig00087, whole genome shot... (27% of genes show similarity)Glarea lozoyensis ATCC 20868 chromosome Unknown GLAREA2, whole... (16% of genes show similarity)Glomerella graminicola M1.001 genomic scaffold supercont1.26, ... (16% of genes show similarity)Colletotrichum orbiculare MAFF 240422 unplaced genomic scaffol... (16% of genes show similarity)Rasamsonia emersonii CBS 393.64 ctg1118621760286, whole g... (16% of genes show similarity)Download graphic

### scaffold\_5 - Cluster 16 - Other

### Predicted core structure

Rough prediction of core scaffold based on assumed PKS/NRPS colinearity; tailoring reactions not taken into account

### Prediction details

Monomers prediction:
:   N/A

### Gene cluster description

Download cluster GenBank file

scaffold\_5 - Gene Cluster 16. Type = other. Location: 2091022 - 2134347 nt. Click on genes for more information.

Show pHMM detection rules used

other: ((PP-binding & AMP-binding) or (PP-binding & A-OX) or (NAD\_binding\_4 & A-OX) or (NAD\_binding\_4 & AMP-binding) or LmbU or Neocarzinostat or cycdipepsynth or fom1 or bcpB or frbD or mitE or vlmB or prnB or CaiA or bacilysin or orf2\_PTase)

#### Legend:

core biosynthetic genes

additional biosynthetic genes

transport-related genes

regulatory genes

other genes

cluster extent as predicted by CASSIS

### Detailed annotation

### Homologous gene clusters

All hitsOidiodendron maius Zn unplaced genomic scaffold scaffold\_5, wh... (100% of genes show similarity)Penicillium marneffei ATCC 18224 scf\_1105668340906 genomic sca... (75% of genes show similarity)Talaromyces stipitatus ATCC 10500 scf\_1105507295515 genomic sc... (66% of genes show similarity)Talaromyces marneffei PM1 scf025, whole genome shotgun seq... (58% of genes show similarity)Claviceps purpurea 20.1 WGS project CAGA00000000 data, con... (41% of genes show similarity)Penicillium solitum strain RS1 scf7180000043182, whole gen... (16% of genes show similarity)Botryotinia fuckeliana T4 SuperContig\_58\_1 genomic supercontig. (16% of genes show similarity)Botryotinia fuckeliana BcDW1 unplaced genomic scaffold Scaffol... (16% of genes show similarity)Fusarium avenaceum strain Fa05001 Fave\_05001\_scaffold23.1,... (16% of genes show similarity)Glomerella graminicola M1.001 genomic scaffold supercont1.43,... (16% of genes show similarity)Download graphic

### scaffold\_5 - Cluster 17 - Other

### Predicted core structure

Rough prediction of core scaffold based on assumed PKS/NRPS colinearity; tailoring reactions not taken into account

### Prediction details

Monomers prediction:
:   N/A

### Gene cluster description

Download cluster GenBank file

scaffold\_5 - Gene Cluster 17. Type = other. Location: 2205493 - 2250549 nt. Click on genes for more information.

Show pHMM detection rules used

other: ((PP-binding & AMP-binding) or (PP-binding & A-OX) or (NAD\_binding\_4 & A-OX) or (NAD\_binding\_4 & AMP-binding) or LmbU or Neocarzinostat or cycdipepsynth or fom1 or bcpB or frbD or mitE or vlmB or prnB or CaiA or bacilysin or orf2\_PTase)

#### Legend:

core biosynthetic genes

additional biosynthetic genes

transport-related genes

regulatory genes

other genes

cluster extent as predicted by CASSIS

### Detailed annotation

### Homologous gene clusters

All hitsOidiodendron maius Zn unplaced genomic scaffold scaffold\_5, wh... (100% of genes show similarity)Aspergillus kawachii IFO 4308 DNA, contig: scaffold00038, whol... (12% of genes show similarity)Neurospora tetrasperma FGSC 2509 unplaced genomic scaffold NEU... (18% of genes show similarity)Neurospora tetrasperma FGSC 2508 unplaced genomic scaffold NEU... (18% of genes show similarity)Fusarium verticillioides 7600 chromosome 1 supercont3.1, whole... (18% of genes show similarity)Jaapia argillacea MUCL 33604 unplaced genomic scaffold JAAARsc... (12% of genes show similarity)Pseudogymnoascus sp. VKM F-4516 (FW-969) contig\_766, whole... (18% of genes show similarity)Fonsecaea pedrosoi CBS 271.37 unplaced genomic scaffold superc... (12% of genes show similarity)Fonsecaea pedrosoi CBS 271.37 unplaced genomic scaffold superc... (18% of genes show similarity)Fusarium fujikuroi strain KSU X-10626 seq\_122, whole geno... (12% of genes show similarity)Download graphic

### scaffold\_6 - Cluster 18 - Terpene

### Gene cluster description

Download cluster GenBank file

scaffold\_6 - Gene Cluster 18. Type = terpene. Location: 1916775 - 1938381 nt. Click on genes for more information.

Show pHMM detection rules used

terpene: (Terpene\_synth or Terpene\_synth\_C or phytoene\_synt or Lycopene\_cycl or terpene\_cyclase or NapT7 or fung\_ggpps or fung\_ggpps2 or trichodiene\_synth or TRI5)

#### Legend:

core biosynthetic genes

additional biosynthetic genes

transport-related genes

regulatory genes

other genes

cluster extent as predicted by CASSIS

### Homologous gene clusters

All hitsOidiodendron maius Zn unplaced genomic scaffold scaffold\_6, wh... (55% of genes show similarity)Fusarium oxysporum f. sp. conglutinans race 2 54008 unplaced g... (25% of genes show similarity)Fusarium oxysporum f. sp. pisi HDV247 unplaced genomic scaffol... (25% of genes show similarity)Fusarium oxysporum f. sp. melonis 26406 unplaced genomic scaff... (25% of genes show similarity)Fusarium oxysporum FOSC 3-a unplaced genomic scaffold supercon... (25% of genes show similarity)Fusarium oxysporum f. sp. lycopersici MN25 unplaced genomic sc... (25% of genes show similarity)Fusarium oxysporum f. sp. radicis-lycopersici 26381 unplaced g... (25% of genes show similarity)Fusarium oxysporum f. sp. lycopersici 4287 supercont2.6 genomi... (25% of genes show similarity)Marssonina brunnea f. sp. 'multigermtubi' MB\_m1 unplaced genom... (25% of genes show similarity)Togninia minima UCRPA7 unplaced genomic scaffold PA7\_03\_scaff... (25% of genes show similarity)Download graphic

### scaffold\_6 - Cluster 19 - T1pks

### Predicted core structure

Rough prediction of core scaffold based on assumed PKS/NRPS colinearity; tailoring reactions not taken into account

### Prediction details

Monomers prediction:
:   (pk)

jgi.p\_Oidma1\_197601
:   PKS signature: mal
:   Minowa: mmal
:   consensus: pk

### Gene cluster description

Download cluster GenBank file

scaffold\_6 - Gene Cluster 19. Type = t1pks. Location: 2041350 - 2088122 nt. Click on genes for more information.

Show pHMM detection rules used

t1pks: ((PKS\_KS & PKS\_AT) or (ene\_KS & PKS\_AT) or (mod\_KS & PKS\_AT) or (hyb\_KS & PKS\_AT) or (itr\_KS & PKS\_AT) or (tra\_KS & PKS\_AT))

#### Legend:

core biosynthetic genes

additional biosynthetic genes

transport-related genes

regulatory genes

other genes

cluster extent as predicted by CASSIS

### Detailed annotation

### Homologous gene clusters

All hitsOidiodendron maius Zn unplaced genomic scaffold scaffold\_6, wh... (100% of genes show similarity)Marssonina brunnea f. sp. 'multigermtubi' MB\_m1 unplaced genom... (25% of genes show similarity)Glarea lozoyensis ATCC 20868 chromosome Unknown GLAREA20, whol... (25% of genes show similarity)Thielavia terrestris NRRL 8126 chromosome 1, complete sequence. (25% of genes show similarity)Sordaria macrospora k-hell, WGS project CABT00000000 data,... (25% of genes show similarity)Magnaporthe poae ATCC 64411 unplaced genomic scaffold supercon... (25% of genes show similarity)Stachybotrys chartarum IBT 40293 unplaced genomic scaffold sca... (25% of genes show similarity)Stachybotrys chartarum IBT 7711 unplaced genomic scaffold scaf... (25% of genes show similarity)Stachybotrys chartarum IBT 40288 unplaced genomic scaffold sca... (25% of genes show similarity)Diaporthe ampelina DA\_912\_scaffold\_v01\_389, whole genome ... (25% of genes show similarity)Download graphic

### scaffold\_8 - Cluster 20 - Nrps

### Predicted core structure

Rough prediction of core scaffold based on assumed PKS/NRPS colinearity; tailoring reactions not taken into account

### Prediction details

Monomers prediction:
:   N/A

### Database cross-links

Link to NORINE database query form  
Direct lookup in NORINE database in strict mode // relaxed mode

### Gene cluster description

Download cluster GenBank file

scaffold\_8 - Gene Cluster 20. Type = nrps. Location: 612777 - 667410 nt. Click on genes for more information.

Show pHMM detection rules used

nrps: ((Condensation & AMP-binding) or (Condensation & A-OX) or cluster(Condensation,AMP-binding))

#### Legend:

core biosynthetic genes

additional biosynthetic genes

transport-related genes

regulatory genes

other genes

cluster extent as predicted by CASSIS

### Detailed annotation

### Homologous gene clusters

All hitsOidiodendron maius Zn unplaced genomic scaffold scaffold\_8, wh... (100% of genes show similarity)Marssonina brunnea f. sp. 'multigermtubi' MB\_m1 unplaced genom... (58% of genes show similarity)Botryotinia fuckeliana BcDW1 unplaced genomic scaffold Scaffol... (50% of genes show similarity)Sclerotinia borealis F-4157 contig00289, whole genome shot... (50% of genes show similarity)Botryotinia fuckeliana T4 SuperContig\_379\_1 genomic supercontig. (66% of genes show similarity)Sclerotinia sclerotiorum 1980 scaffold\_4 genomic scaffold, who... (41% of genes show similarity)Erysiphe necator strain c c-strain-scaffold\_4338, whole ge... (33% of genes show similarity)Aureobasidium pullulans var. pullulans EXF-150 unplaced genomi... (25% of genes show similarity)Aureobasidium subglaciale EXF-2481 unplaced genomic scaffold s... (25% of genes show similarity)Aureobasidium pullulans var. melanogenum CBS 110374 unplaced ... (25% of genes show similarity)Download graphic

### scaffold\_8 - Cluster 21 - T1pks

### Predicted core structure

Rough prediction of core scaffold based on assumed PKS/NRPS colinearity; tailoring reactions not taken into account

### Prediction details

Monomers prediction:
:   N/A

### Gene cluster description

Download cluster GenBank file

scaffold\_8 - Gene Cluster 21. Type = t1pks. Location: 1363091 - 1411375 nt. Click on genes for more information.

Show pHMM detection rules used

t1pks: ((PKS\_KS & PKS\_AT) or (ene\_KS & PKS\_AT) or (mod\_KS & PKS\_AT) or (hyb\_KS & PKS\_AT) or (itr\_KS & PKS\_AT) or (tra\_KS & PKS\_AT))

#### Legend:

core biosynthetic genes

additional biosynthetic genes

transport-related genes

regulatory genes

other genes

cluster extent as predicted by CASSIS

### Detailed annotation

### Homologous gene clusters

All hitsOidiodendron maius Zn unplaced genomic scaffold scaffold\_8, wh... (100% of genes show similarity)Pseudogymnoascus sp. VKM F-4517 (FW-2822) contig\_47, whole... (17% of genes show similarity)Pseudogymnoascus sp. VKM F-4515 (FW-2607) contig\_93, whole... (17% of genes show similarity)Talaromyces stipitatus ATCC 10500 scf\_1105507295511 genomic sc... (23% of genes show similarity)Exophiala aquamarina CBS 119918 aczIA-supercont1.7, whole ... (23% of genes show similarity)Trichoderma harzianum strain T6776 Scaffolds0086.1, whole ... (17% of genes show similarity)Trichoderma virens Gv29-8 TRIVIscaffold\_52, whole genome s... (17% of genes show similarity)Rasamsonia emersonii CBS 393.64 ctg1118621759978, whole ge... (23% of genes show similarity)Talaromyces islandicus genome assembly PIS, scaffold WF-38... (23% of genes show similarity)Bipolaris oryzae ATCC 44560 unplaced genomic scaffold scaffol... (17% of genes show similarity)Download graphic

### scaffold\_9 - Cluster 22 - T1pks

### Predicted core structure

Rough prediction of core scaffold based on assumed PKS/NRPS colinearity; tailoring reactions not taken into account

### Prediction details

Monomers prediction:
:   N/A

### Gene cluster description

Download cluster GenBank file

scaffold\_9 - Gene Cluster 22. Type = t1pks. Location: 739406 - 787913 nt. Click on genes for more information.

Show pHMM detection rules used

t1pks: ((PKS\_KS & PKS\_AT) or (ene\_KS & PKS\_AT) or (mod\_KS & PKS\_AT) or (hyb\_KS & PKS\_AT) or (itr\_KS & PKS\_AT) or (tra\_KS & PKS\_AT))

#### Legend:

core biosynthetic genes

additional biosynthetic genes

transport-related genes

regulatory genes

other genes

cluster extent as predicted by CASSIS

### Detailed annotation

### Homologous gene clusters

All hitsOidiodendron maius Zn unplaced genomic scaffold scaffold\_9, wh... (100% of genes show similarity)Rasamsonia emersonii CBS 393.64 ctg1118621759978, whole ge... (16% of genes show similarity)Talaromyces islandicus genome assembly PIS, scaffold WF-38... (16% of genes show similarity)Gaeumannomyces graminis var. tritici R3-111a-1 unplaced genomi... (16% of genes show similarity)Oidiodendron maius Zn unplaced genomic scaffold scaffold\_20, w... (11% of genes show similarity)Pseudogymnoascus sp. VKM F-4515 (FW-2607) contig\_93, whole... (16% of genes show similarity)Pseudogymnoascus sp. VKM F-4517 (FW-2822) contig\_47, whole... (16% of genes show similarity)Oidiodendron maius Zn unplaced genomic scaffold scaffold\_8, wh... (16% of genes show similarity)Neofusicoccum parvum UCRNP2 chromosome Unknown NP2\_03\_scaffold... (11% of genes show similarity)Diaporthe ampelina DA\_912\_scaffold\_v01\_11, whole genome s... (11% of genes show similarity)Download graphic

### scaffold\_9 - Cluster 23 - T1pks

### Predicted core structure

Rough prediction of core scaffold based on assumed PKS/NRPS colinearity; tailoring reactions not taken into account

### Prediction details

Monomers prediction:
:   N/A

### Gene cluster description

Download cluster GenBank file

scaffold\_9 - Gene Cluster 23. Type = t1pks. Location: 937413 - 985702 nt. Click on genes for more information.

Show pHMM detection rules used

t1pks: ((PKS\_KS & PKS\_AT) or (ene\_KS & PKS\_AT) or (mod\_KS & PKS\_AT) or (hyb\_KS & PKS\_AT) or (itr\_KS & PKS\_AT) or (tra\_KS & PKS\_AT))

#### Legend:

core biosynthetic genes

additional biosynthetic genes

transport-related genes

regulatory genes

other genes

cluster extent as predicted by CASSIS

### Detailed annotation

### Homologous gene clusters

All hitsOidiodendron maius Zn unplaced genomic scaffold scaffold\_9, wh... (100% of genes show similarity)Fusarium oxysporum f. sp. melonis 26406 unplaced genomic scaff... (23% of genes show similarity)Nectria haematococca mpVI 77-13-4 chromosome 7 genomic scaffol... (23% of genes show similarity)Fusarium oxysporum f. sp. lycopersici 4287 supercont2.31 genom... (23% of genes show similarity)Fusarium acuminatum CS5907 WGS project CBMG000000000 data... (23% of genes show similarity)Fusarium oxysporum Fo5176 contig05962, whole genome shotgu... (23% of genes show similarity)Fusarium oxysporum f. sp. lycopersici 4287 supercont2.25 genom... (23% of genes show similarity)Neonectria ditissima strain R09/05 contig\_42, whole genome... (15% of genes show similarity)Fusarium fujikuroi strain KSU X-10626 seq\_79, whole genome... (15% of genes show similarity)Fusarium fujikuroi strain FGSC 8932 seq\_289, whole genome... (15% of genes show similarity)Download graphic

### scaffold\_9 - Cluster 24 - T1pks

### Predicted core structure

Rough prediction of core scaffold based on assumed PKS/NRPS colinearity; tailoring reactions not taken into account

### Prediction details

Monomers prediction:
:   N/A

### Gene cluster description

Download cluster GenBank file

scaffold\_9 - Gene Cluster 24. Type = t1pks. Location: 1072255 - 1134365 nt. Click on genes for more information.

Show pHMM detection rules used

t1pks: ((PKS\_KS & PKS\_AT) or (ene\_KS & PKS\_AT) or (mod\_KS & PKS\_AT) or (hyb\_KS & PKS\_AT) or (itr\_KS & PKS\_AT) or (tra\_KS & PKS\_AT))

#### Legend:

core biosynthetic genes

additional biosynthetic genes

transport-related genes

regulatory genes

other genes

cluster extent as predicted by CASSIS

### Detailed annotation

### Homologous gene clusters

All hitsOidiodendron maius Zn unplaced genomic scaffold scaffold\_9, wh... (100% of genes show similarity)Actinomadura kijaniata fructokinase gene, partial cds; kijanim... (8% of genes show similarity)Streptomyces sp. SCSIO 01127 macrolide glycosyltransferase and... (8% of genes show similarity)Cochliobolus sativus ND90Pr unplaced genomic scaffold COCSAsca... (12% of genes show similarity)Talaromyces stipitatus ATCC 10500 scf\_1105507295517 genomic sc... (12% of genes show similarity)Fusarium avenaceum strain Fa05001 Fave\_05001\_scaffold23.1,... (12% of genes show similarity)Pseudonocardia sp. AL041005-10, complete genome. (8% of genes show similarity)Streptomyces sp. FXJ7.023 lobophorin biosynthetic gene cluster... (8% of genes show similarity)Actinoplanes sp. N902-109, complete genome. (29% of genes show similarity)Talaromyces islandicus genome assembly PIS, scaffold WF-... (12% of genes show similarity)Download graphic

### scaffold\_9 - Cluster 25 - T1pks

### Predicted core structure

Rough prediction of core scaffold based on assumed PKS/NRPS colinearity; tailoring reactions not taken into account

### Prediction details

Monomers prediction:
:   N/A

### Gene cluster description

Download cluster GenBank file

scaffold\_9 - Gene Cluster 25. Type = t1pks. Location: 1326212 - 1371648 nt. Click on genes for more information.

Show pHMM detection rules used

t1pks: ((PKS\_KS & PKS\_AT) or (ene\_KS & PKS\_AT) or (mod\_KS & PKS\_AT) or (hyb\_KS & PKS\_AT) or (itr\_KS & PKS\_AT) or (tra\_KS & PKS\_AT))

#### Legend:

core biosynthetic genes

additional biosynthetic genes

transport-related genes

regulatory genes

other genes

cluster extent as predicted by CASSIS

### Detailed annotation

### Homologous gene clusters

All hitsOidiodendron maius Zn unplaced genomic scaffold scaffold\_9, wh... (100% of genes show similarity)Aspergillus fumigatus Z5 chromosome Unknown scaffold\_14, whole... (27% of genes show similarity)Aspergillus fumigatus A1163 scf\_000008 genomic scaffold, whole... (27% of genes show similarity)Aspergillus fumigatus Af293 chromosome 7 afu1\_103, whole g... (27% of genes show similarity)Fusarium verticillioides 7600 chromosome 5 supercont3.3, whole... (27% of genes show similarity)Talaromyces cellulolyticus DNA, scaffold: scf\_tce0\_034, strain... (18% of genes show similarity)Fusarium oxysporum f. sp. lycopersici 4287 supercont2.5 genomi... (18% of genes show similarity)Aspergillus fumigatus var. RP-2014 strain niveus afvn\_516,... (18% of genes show similarity)Aspergillus ochraceoroseus strain SRRC1432 contig00354, wh... (18% of genes show similarity)Aspergillus rambellii strain SRRC1468 contig00183, whole ... (18% of genes show similarity)Download graphic

### scaffold\_10 - Cluster 26 - Other

### Predicted core structure

Rough prediction of core scaffold based on assumed PKS/NRPS colinearity; tailoring reactions not taken into account

### Prediction details

Monomers prediction:
:   N/A

### Gene cluster description

Download cluster GenBank file

scaffold\_10 - Gene Cluster 26. Type = other. Location: 137993 - 181568 nt. Click on genes for more information.

Show pHMM detection rules used

other: ((PP-binding & AMP-binding) or (PP-binding & A-OX) or (NAD\_binding\_4 & A-OX) or (NAD\_binding\_4 & AMP-binding) or LmbU or Neocarzinostat or cycdipepsynth or fom1 or bcpB or frbD or mitE or vlmB or prnB or CaiA or bacilysin or orf2\_PTase)

#### Legend:

core biosynthetic genes

additional biosynthetic genes

transport-related genes

regulatory genes

other genes

cluster extent as predicted by CASSIS

### Detailed annotation

### Homologous gene clusters

All hitsOidiodendron maius Zn unplaced genomic scaffold scaffold\_10, w... (100% of genes show similarity)Glarea lozoyensis ATCC 20868 chromosome Unknown GLAREA18, whol... (36% of genes show similarity)Emmonsia crescens UAMH 3008 supercont1.952, whole genome s... (36% of genes show similarity)Ajellomyces dermatitidis ER-3 genomic scaffold supercont1.6, w... (36% of genes show similarity)Ajellomyces dermatitidis SLH14081 genomic scaffold supercont1.... (36% of genes show similarity)Paracoccidioides sp. 'lutzii' Pb01 unplaced genomic scaffold s... (36% of genes show similarity)Aureobasidium pullulans var. pullulans EXF-150 unplaced genomi... (15% of genes show similarity)Baudoinia panamericana UAMH 10762 unplaced genomic scaffold BA... (15% of genes show similarity)Paracoccidioides brasiliensis Pb18 unplaced genomic scaffold s... (31% of genes show similarity)Paracoccidioides brasiliensis Pb03 unplaced genomic scaffold ... (31% of genes show similarity)Download graphic

### scaffold\_11 - Cluster 27 - T3pks-t1pks

### Predicted core structure

Rough prediction of core scaffold based on assumed PKS/NRPS colinearity; tailoring reactions not taken into account

### Prediction details

Monomers prediction:
:   N/A

### Gene cluster description

Download cluster GenBank file

scaffold\_11 - Gene Cluster 27. Type = t3pks-t1pks. Location: 1184930 - 1238558 nt. Click on genes for more information.

Show pHMM detection rules used

t3pks: (Chal\_sti\_synt\_C or Chal\_sti\_synt\_N)  
t1pks: ((PKS\_KS & PKS\_AT) or (ene\_KS & PKS\_AT) or (mod\_KS & PKS\_AT) or (hyb\_KS & PKS\_AT) or (itr\_KS & PKS\_AT) or (tra\_KS & PKS\_AT))

#### Legend:

core biosynthetic genes

additional biosynthetic genes

transport-related genes

regulatory genes

other genes

cluster extent as predicted by CASSIS

### Detailed annotation

### Homologous gene clusters

All hitsOidiodendron maius Zn unplaced genomic scaffold scaffold\_11, w... (100% of genes show similarity)Glomerella graminicola M1.001 genomic scaffold supercont1.45, ... (16% of genes show similarity)Exophiala spinifera strain CBS 89968 unplaced genomic scaffold... (16% of genes show similarity)Colletotrichum gloeosporioides Nara gc5 unplaced genomic scaff... (16% of genes show similarity)Podospora anserina genomic DNA, chromosome 6. (11% of genes show similarity)Podospora anserina S mat+ genomic DNA chromosome 6, superconti... (11% of genes show similarity)Nectria haematococca mpVI 77-13-4 chromosome 3 genomic scaffol... (11% of genes show similarity)Beauveria bassiana ARSEF 2860 unplaced genomic scaffold BBA\_S0... (11% of genes show similarity)Parastagonospora nodorum SN15 scaffold\_15, whole genome shotgu... (11% of genes show similarity)Fusarium avenaceum strain Fa05001 Fave\_05001\_scaffold12.1... (11% of genes show similarity)Download graphic

### scaffold\_12 - Cluster 28 - T1pks

### Predicted core structure

Rough prediction of core scaffold based on assumed PKS/NRPS colinearity; tailoring reactions not taken into account

### Prediction details

Monomers prediction:
:   (mal)

jgi.p\_Oidma1\_182120
:   PKS signature: mal
:   Minowa: mal
:   consensus: mal

### Gene cluster description

Download cluster GenBank file

scaffold\_12 - Gene Cluster 28. Type = t1pks. Location: 373442 - 421357 nt. Click on genes for more information.

Show pHMM detection rules used

t1pks: ((PKS\_KS & PKS\_AT) or (ene\_KS & PKS\_AT) or (mod\_KS & PKS\_AT) or (hyb\_KS & PKS\_AT) or (itr\_KS & PKS\_AT) or (tra\_KS & PKS\_AT))

#### Legend:

core biosynthetic genes

additional biosynthetic genes

transport-related genes

regulatory genes

other genes

cluster extent as predicted by CASSIS

### Detailed annotation

### Homologous gene clusters

All hitsOidiodendron maius Zn unplaced genomic scaffold scaffold\_12, w... (100% of genes show similarity)Aspergillus ochraceoroseus strain SRRC1432 contig00009, wh... (14% of genes show similarity)Aspergillus rambellii strain SRRC1468 contig00102, whole g... (14% of genes show similarity)Aspergillus ustus strain 3.3904 AUSC\_69, whole genome shot... (14% of genes show similarity)Glarea lozoyensis ATCC 20868 chromosome Unknown GLAREA5, whole... (21% of genes show similarity)Arthroderma gypseum CBS 118893 supercont1.7 genomic scaffold, ... (14% of genes show similarity)Trichophyton interdigitale H6 unplaced genomic scaffold superc... (14% of genes show similarity)Trichophyton interdigitale MR816 cont1.211, whole genome s... (14% of genes show similarity)Trichophyton tonsurans CBS 112818 genomic scaffold supercont1.... (14% of genes show similarity)Trichophyton equinum CBS 127.97 supercont1.15 genomic scaffol... (14% of genes show similarity)Download graphic

### scaffold\_13 - Cluster 29 - T1pks

### Predicted core structure

Rough prediction of core scaffold based on assumed PKS/NRPS colinearity; tailoring reactions not taken into account

### Prediction details

Monomers prediction:
:   N/A

### Gene cluster description

Download cluster GenBank file

scaffold\_13 - Gene Cluster 29. Type = t1pks. Location: 32112 - 79801 nt. Click on genes for more information.

Show pHMM detection rules used

t1pks: ((PKS\_KS & PKS\_AT) or (ene\_KS & PKS\_AT) or (mod\_KS & PKS\_AT) or (hyb\_KS & PKS\_AT) or (itr\_KS & PKS\_AT) or (tra\_KS & PKS\_AT))

#### Legend:

core biosynthetic genes

additional biosynthetic genes

transport-related genes

regulatory genes

other genes

cluster extent as predicted by CASSIS

### Detailed annotation

### Homologous gene clusters

All hitsOidiodendron maius Zn unplaced genomic scaffold scaffold\_13, w... (100% of genes show similarity)Pseudocercospora fijiensis CIRAD86 unplaced genomic scaffold M... (14% of genes show similarity)Penicillium solitum strain RS1 scf7180000043670, whole gen... (14% of genes show similarity)Colletotrichum gloeosporioides Cg-14 contig02240, whole ge... (14% of genes show similarity)Colletotrichum gloeosporioides Nara gc5 unplaced genomic scaff... (14% of genes show similarity)Mycosphaerella graminicola IPO323 chromosome 1, whole genome s... (14% of genes show similarity)Zymoseptoria brevis strain Zb18110 contig4104, whole genom... (14% of genes show similarity)Neonectria ditissima strain R09/05 contig\_101, whole genom... (14% of genes show similarity)Colletotrichum gloeosporioides Nara gc5 unplaced genomic scaff... (21% of genes show similarity)Myceliophthora thermophila ATCC 42464 chromosome 3, complete ... (21% of genes show similarity)Download graphic

### scaffold\_13 - Cluster 30 - T1pks

### Predicted core structure

Rough prediction of core scaffold based on assumed PKS/NRPS colinearity; tailoring reactions not taken into account

### Prediction details

Monomers prediction:
:   N/A

### Gene cluster description

Download cluster GenBank file

scaffold\_13 - Gene Cluster 30. Type = t1pks. Location: 246219 - 293290 nt. Click on genes for more information.

Show pHMM detection rules used

t1pks: ((PKS\_KS & PKS\_AT) or (ene\_KS & PKS\_AT) or (mod\_KS & PKS\_AT) or (hyb\_KS & PKS\_AT) or (itr\_KS & PKS\_AT) or (tra\_KS & PKS\_AT))

#### Legend:

core biosynthetic genes

additional biosynthetic genes

transport-related genes

regulatory genes

other genes

cluster extent as predicted by CASSIS

### Detailed annotation

### Homologous gene clusters

All hitsOidiodendron maius Zn unplaced genomic scaffold scaffold\_13, w... (100% of genes show similarity)Acremonium chrysogenum ATCC 11550 scaffold54, whole genome... (33% of genes show similarity)Eutypa lata UCREL1 unplaced genomic scaffold EL1\_03\_scaffold\_2... (33% of genes show similarity)Aureobasidium pullulans var. pullulans EXF-150 unplaced genomi... (16% of genes show similarity)Magnaporthe oryzae P131 unplaced genomic scaffold P131\_scaffol... (16% of genes show similarity)Aspergillus flavus AF70 1, whole genome shotgun sequence. (16% of genes show similarity)Aspergillus oryzae 3.042 Contig181, whole genome shotgun s... (16% of genes show similarity)Aspergillus oryzae 100-8 Contig161.4, whole genome shotgun... (16% of genes show similarity)Aspergillus oryzae RIB40 DNA, SC020. (16% of genes show similarity)Glarea lozoyensis ATCC 20868 chromosome Unknown GLAREA20, who... (11% of genes show similarity)Download graphic

### scaffold\_13 - Cluster 31 - Nrps

### Predicted core structure

Rough prediction of core scaffold based on assumed PKS/NRPS colinearity; tailoring reactions not taken into account

### Prediction details

Monomers prediction:
:   N/A

### Database cross-links

Link to NORINE database query form  
Direct lookup in NORINE database in strict mode // relaxed mode

### Gene cluster description

Download cluster GenBank file

scaffold\_13 - Gene Cluster 31. Type = nrps. Location: 506748 - 554021 nt. Click on genes for more information.

Show pHMM detection rules used

nrps: ((Condensation & AMP-binding) or (Condensation & A-OX) or cluster(Condensation,AMP-binding))

#### Legend:

core biosynthetic genes

additional biosynthetic genes

transport-related genes

regulatory genes

other genes

cluster extent as predicted by CASSIS

### Detailed annotation

### Homologous gene clusters

All hitsOidiodendron maius Zn unplaced genomic scaffold scaffold\_13, w... (100% of genes show similarity)Trichoderma virens Gv29-8 TRIVIscaffold\_85, whole genome s... (12% of genes show similarity)Trichoderma atroviride IMI 206040 TRIATcontig\_23, whole ge... (12% of genes show similarity)Trichoderma harzianum strain T6776 Scaffolds0058.1, whole ... (12% of genes show similarity)Oidiodendron maius Zn unplaced genomic scaffold scaffold\_17, w... (12% of genes show similarity)Penicillium digitatum Pd1 contig270, whole genome shotgun ... (12% of genes show similarity)Penicillium digitatum PHI26 contig37, whole genome shotgun... (12% of genes show similarity)Aspergillus rambellii strain SRRC1468 contig00499, whole g... (12% of genes show similarity)Aspergillus ochraceoroseus strain SRRC1432 contig00188, wh... (12% of genes show similarity)Aspergillus niger contig An16c0200, genomic contig. (12% of genes show similarity)Download graphic

### scaffold\_13 - Cluster 32 - T1pks-nrps

### Predicted core structure

Rough prediction of core scaffold based on assumed PKS/NRPS colinearity; tailoring reactions not taken into account

### Prediction details

Monomers prediction:
:   N/A

### Database cross-links

Link to NORINE database query form  
Direct lookup in NORINE database in strict mode // relaxed mode

### Gene cluster description

Download cluster GenBank file

scaffold\_13 - Gene Cluster 32. Type = t1pks-nrps. Location: 732574 - 784914 nt. Click on genes for more information.

Show pHMM detection rules used

t1pks: ((PKS\_KS & PKS\_AT) or (ene\_KS & PKS\_AT) or (mod\_KS & PKS\_AT) or (hyb\_KS & PKS\_AT) or (itr\_KS & PKS\_AT) or (tra\_KS & PKS\_AT))  
nrps: ((Condensation & AMP-binding) or (Condensation & A-OX) or cluster(Condensation,AMP-binding))

#### Legend:

core biosynthetic genes

additional biosynthetic genes

transport-related genes

regulatory genes

other genes

cluster extent as predicted by CASSIS

### Detailed annotation

### Homologous gene clusters

All hitsOidiodendron maius Zn unplaced genomic scaffold scaffold\_13, w... (100% of genes show similarity)Penicillium roqueforti FM164 genomic scaffold, ProqFM164S02, w... (8% of genes show similarity)Macrophomina phaseolina MS6 contig00324, whole genome shot... (13% of genes show similarity)Fusarium heterosporum strain ATCC 74349 fusaridione A gene clu... (8% of genes show similarity)Fusarium oxysporum f. sp. pisi HDV247 unplaced genomic scaffol... (8% of genes show similarity)Fusarium oxysporum f. sp. conglutinans race 2 54008 unplaced g... (8% of genes show similarity)Fusarium oxysporum Fo5176 contig00183, whole genome shotgu... (8% of genes show similarity)Fusarium oxysporum f. sp. raphani 54005 unplaced genomic scaff... (8% of genes show similarity)Fusarium oxysporum FOSC 3-a unplaced genomic scaffold supercon... (8% of genes show similarity)Fusarium oxysporum f. sp. lycopersici 4287 supercont2.26 geno... (8% of genes show similarity)Download graphic

### Homologous known gene clusters

All hitsFusaridione A biosynthetic gene cluster (12% of genes show similarity)Equisetin biosynthetic gene cluster (12% of genes show similarity)Download graphic

### scaffold\_13 - Cluster 33 - T1pks

### Predicted core structure

Rough prediction of core scaffold based on assumed PKS/NRPS colinearity; tailoring reactions not taken into account

### Prediction details

Monomers prediction:
:   N/A

### Gene cluster description

Download cluster GenBank file

scaffold\_13 - Gene Cluster 33. Type = t1pks. Location: 895311 - 942376 nt. Click on genes for more information.

Show pHMM detection rules used

t1pks: ((PKS\_KS & PKS\_AT) or (ene\_KS & PKS\_AT) or (mod\_KS & PKS\_AT) or (hyb\_KS & PKS\_AT) or (itr\_KS & PKS\_AT) or (tra\_KS & PKS\_AT))

#### Legend:

core biosynthetic genes

additional biosynthetic genes

transport-related genes

regulatory genes

other genes

cluster extent as predicted by CASSIS

### Detailed annotation

### Homologous gene clusters

All hitsOidiodendron maius Zn unplaced genomic scaffold scaffold\_13, w... (100% of genes show similarity)Thielavia terrestris NRRL 8126 chromosome 3, complete sequence. (26% of genes show similarity)Talaromyces cellulolyticus DNA, scaffold: scf\_tce0\_041, strain... (20% of genes show similarity)Penicillium digitatum PHI26 contig270, whole genome shotgu... (13% of genes show similarity)Penicillium digitatum Pd1 contig322, whole genome shotgun ... (13% of genes show similarity)Aspergillus fumigatus Z5 chromosome Unknown scaffold\_8, whole ... (20% of genes show similarity)Talaromyces marneffei PM1 scf002, whole genome shotgun seq... (20% of genes show similarity)Metarhizium brunneum ARSEF 3297 scaffold\_8, whole genome s... (20% of genes show similarity)Metarhizium guizhouense ARSEF 977 scaffold\_48, whole genom... (20% of genes show similarity)Neosartorya udagawae DNA, contig:Aud0116, strain: IFM 469... (20% of genes show similarity)Download graphic

### scaffold\_14 - Cluster 34 - T1pks

### Predicted core structure

Rough prediction of core scaffold based on assumed PKS/NRPS colinearity; tailoring reactions not taken into account

### Prediction details

Monomers prediction:
:   N/A

### Gene cluster description

Download cluster GenBank file

scaffold\_14 - Gene Cluster 34. Type = t1pks. Location: 563359 - 610042 nt. Click on genes for more information.

Show pHMM detection rules used

t1pks: ((PKS\_KS & PKS\_AT) or (ene\_KS & PKS\_AT) or (mod\_KS & PKS\_AT) or (hyb\_KS & PKS\_AT) or (itr\_KS & PKS\_AT) or (tra\_KS & PKS\_AT))

#### Legend:

core biosynthetic genes

additional biosynthetic genes

transport-related genes

regulatory genes

other genes

cluster extent as predicted by CASSIS

### Detailed annotation

### Homologous gene clusters

All hitsOidiodendron maius Zn unplaced genomic scaffold scaffold\_14, w... (100% of genes show similarity)Arthroderma gypseum CBS 118893 supercont1.10 genomic scaffold,... (21% of genes show similarity)Myceliophthora thermophila ATCC 42464 chromosome 3, complete s... (15% of genes show similarity)Neosartorya fischeri NRRL 181 1099437636245 genomic scaffold, ... (15% of genes show similarity)Glomerella graminicola M1.001 genomic scaffold supercont1.42, ... (15% of genes show similarity)Aspergillus ustus strain 3.3904 AUSC\_33, whole genome shot... (15% of genes show similarity)Coniosporium apollinis CBS 100218 chromosome Unknown supercont... (10% of genes show similarity)Pseudogymnoascus sp. VKM F-4518 (FW-2643) contig\_3667, who... (10% of genes show similarity)Coniosporium apollinis CBS 100218 chromosome Unknown supercont... (10% of genes show similarity)Fonsecaea pedrosoi CBS 271.37 unplaced genomic scaffold super... (10% of genes show similarity)Download graphic

### scaffold\_14 - Cluster 35 - Indole-t1pks

### Predicted core structure

Rough prediction of core scaffold based on assumed PKS/NRPS colinearity; tailoring reactions not taken into account

### Prediction details

Monomers prediction:
:   N/A

### Gene cluster description

Download cluster GenBank file

scaffold\_14 - Gene Cluster 35. Type = indole-t1pks. Location: 719773 - 767883 nt. Click on genes for more information.

Show pHMM detection rules used

indole: (indsynth or dmat or indole\_PTase)  
t1pks: ((PKS\_KS & PKS\_AT) or (ene\_KS & PKS\_AT) or (mod\_KS & PKS\_AT) or (hyb\_KS & PKS\_AT) or (itr\_KS & PKS\_AT) or (tra\_KS & PKS\_AT))

#### Legend:

core biosynthetic genes

additional biosynthetic genes

transport-related genes

regulatory genes

other genes

cluster extent as predicted by CASSIS

### Detailed annotation

### Homologous gene clusters

All hitsOidiodendron maius Zn unplaced genomic scaffold scaffold\_14, w... (100% of genes show similarity)Neosartorya fischeri NRRL 181 1099437636259 genomic scaffold, ... (25% of genes show similarity)Aspergillus fumigatus var. RP-2014 strain niveus afvn\_516,... (25% of genes show similarity)Aspergillus fumigatus Z5 chromosome Unknown scaffold\_14, whole... (25% of genes show similarity)Aspergillus fumigatus A1163 scf\_000008 genomic scaffold, whole... (25% of genes show similarity)Aspergillus fumigatus Af293 chromosome 7 afu1\_103, whole g... (25% of genes show similarity)Microsporum canis CBS 113480 supercont1.3 genomic scaffold, wh... (25% of genes show similarity)Aspergillus lentulus DNA, contig: Alt08, strain: IFM 54703... (25% of genes show similarity)Arthroderma gypseum CBS 118893 supercont1.6 genomic scaffold, ... (25% of genes show similarity)Trichophyton rubrum MR850 unplaced genomic scaffold supercont... (25% of genes show similarity)Download graphic

### Homologous known gene clusters

All hitsNeosartoricin biosynthetic gene cluster (60% of genes show similarity)Viridicatumtoxin biosynthetic gene cluster (60% of genes show similarity)TAN-1612 biosynthetic gene cluster (60% of genes show similarity)Asperthecin biosynthetic gene cluster (40% of genes show similarity)Emericellin biosynthetic gene cluster (40% of genes show similarity)Trypacidin biosynthetic gene cluster (40% of genes show similarity)Pestheic acid biosynthetic gene cluster (40% of genes show similarity)Alternariol biosynthetic gene cluster (40% of genes show similarity)Download graphic

### scaffold\_14 - Cluster 36 - T1pks

### Predicted core structure

Rough prediction of core scaffold based on assumed PKS/NRPS colinearity; tailoring reactions not taken into account

### Prediction details

Monomers prediction:
:   N/A

### Gene cluster description

Download cluster GenBank file

scaffold\_14 - Gene Cluster 36. Type = t1pks. Location: 990688 - 1037285 nt. Click on genes for more information.

Show pHMM detection rules used

t1pks: ((PKS\_KS & PKS\_AT) or (ene\_KS & PKS\_AT) or (mod\_KS & PKS\_AT) or (hyb\_KS & PKS\_AT) or (itr\_KS & PKS\_AT) or (tra\_KS & PKS\_AT))

#### Legend:

core biosynthetic genes

additional biosynthetic genes

transport-related genes

regulatory genes

other genes

cluster extent as predicted by CASSIS

### Detailed annotation

### Homologous gene clusters

All hitsOidiodendron maius Zn unplaced genomic scaffold scaffold\_14, w... (100% of genes show similarity)Talaromyces stipitatus ATCC 10500 scf\_1105507295527 genomic sc... (50% of genes show similarity)Penicillium marneffei ATCC 18224 scf\_1105668340758 genomic sca... (50% of genes show similarity)Talaromyces marneffei PM1 scf026, whole genome shotgun seq... (50% of genes show similarity)Rasamsonia emersonii CBS 393.64 ctg1118621760037, whole ge... (35% of genes show similarity)Parastagonospora nodorum SN15 scaffold\_12, whole genome shotgu... (35% of genes show similarity)Neonectria ditissima strain R09/05 contig\_92, whole genome... (40% of genes show similarity)Aspergillus niger contig An03c0180, genomic contig. (30% of genes show similarity)Aspergillus niger ATCC 1015, whole genome shotgun sequence. (35% of genes show similarity)Endocarpon pusillum Z07020 unplaced genomic scaffold C653857,... (30% of genes show similarity)Download graphic

### scaffold\_15 - Cluster 37 - Other

### Predicted core structure

Rough prediction of core scaffold based on assumed PKS/NRPS colinearity; tailoring reactions not taken into account

### Prediction details

Monomers prediction:
:   N/A

### Gene cluster description

Download cluster GenBank file

scaffold\_15 - Gene Cluster 37. Type = other. Location: 676610 - 719984 nt. Click on genes for more information.

Show pHMM detection rules used

other: ((PP-binding & AMP-binding) or (PP-binding & A-OX) or (NAD\_binding\_4 & A-OX) or (NAD\_binding\_4 & AMP-binding) or LmbU or Neocarzinostat or cycdipepsynth or fom1 or bcpB or frbD or mitE or vlmB or prnB or CaiA or bacilysin or orf2\_PTase)

#### Legend:

core biosynthetic genes

additional biosynthetic genes

transport-related genes

regulatory genes

other genes

cluster extent as predicted by CASSIS

### Detailed annotation

### Homologous gene clusters

All hitsOidiodendron maius Zn unplaced genomic scaffold scaffold\_15, w... (100% of genes show similarity)Botryotinia fuckeliana BcDW1 unplaced genomic scaffold Scaffol... (33% of genes show similarity)Aspergillus nidulans FGSC A4 chromosome III ANcontig1.82, ... (19% of genes show similarity)Sclerotinia borealis F-4157 contig00261, whole genome shot... (33% of genes show similarity)Sclerotinia sclerotiorum 1980 scaffold\_15 genomic scaffold, wh... (28% of genes show similarity)Fusarium oxysporum Fo47 unplaced genomic scaffold supercont1.2... (19% of genes show similarity)Fusarium oxysporum f. sp. radicis-lycopersici 26381 unplaced g... (19% of genes show similarity)Fusarium oxysporum f. sp. lycopersici MN25 unplaced genomic sc... (19% of genes show similarity)Aspergillus ustus strain 3.3904 AUSC\_25, whole genome shot... (28% of genes show similarity)Diplodia seriata DS\_831\_scaffold\_v01\_59, whole genome sho... (19% of genes show similarity)Download graphic

### scaffold\_16 - Cluster 38 - Other

### Predicted core structure

Rough prediction of core scaffold based on assumed PKS/NRPS colinearity; tailoring reactions not taken into account

### Prediction details

Monomers prediction:
:   N/A

### Gene cluster description

Download cluster GenBank file

scaffold\_16 - Gene Cluster 38. Type = other. Location: 221628 - 264778 nt. Click on genes for more information.

Show pHMM detection rules used

other: ((PP-binding & AMP-binding) or (PP-binding & A-OX) or (NAD\_binding\_4 & A-OX) or (NAD\_binding\_4 & AMP-binding) or LmbU or Neocarzinostat or cycdipepsynth or fom1 or bcpB or frbD or mitE or vlmB or prnB or CaiA or bacilysin or orf2\_PTase)

#### Legend:

core biosynthetic genes

additional biosynthetic genes

transport-related genes

regulatory genes

other genes

cluster extent as predicted by CASSIS

### Detailed annotation

### Homologous gene clusters

All hitsOidiodendron maius Zn unplaced genomic scaffold scaffold\_16, w... (100% of genes show similarity)Hydnomerulius pinastri MD-312 unplaced genomic scaffold scaffo... (20% of genes show similarity)Paxillus involutus ATCC 200175 unplaced genomic scaffold PAXIN... (20% of genes show similarity)Oidiodendron maius Zn unplaced genomic scaffold scaffold\_16, w... (13% of genes show similarity)Talaromyces cellulolyticus DNA, scaffold: scf\_tce0\_041, strain... (13% of genes show similarity)Hydnomerulius pinastri MD-312 unplaced genomic scaffold scaffo... (13% of genes show similarity)Pisolithus tinctorius Marx 270 unplaced genomic scaffold scaff... (13% of genes show similarity)Suillus luteus UH-Slu-Lm8-n1 unplaced genomic scaffold CY34sca... (13% of genes show similarity)Togninia minima UCRPA7 unplaced genomic scaffold PA7\_03\_scaffo... (26% of genes show similarity)Nectria haematococca mpVI 77-13-4 chromosome 9 genomic scaffo... (13% of genes show similarity)Download graphic

### scaffold\_16 - Cluster 39 - Other

### Predicted core structure

Rough prediction of core scaffold based on assumed PKS/NRPS colinearity; tailoring reactions not taken into account

### Prediction details

Monomers prediction:
:   N/A

### Gene cluster description

Download cluster GenBank file

scaffold\_16 - Gene Cluster 39. Type = other. Location: 787561 - 830392 nt. Click on genes for more information.

Show pHMM detection rules used

other: ((PP-binding & AMP-binding) or (PP-binding & A-OX) or (NAD\_binding\_4 & A-OX) or (NAD\_binding\_4 & AMP-binding) or LmbU or Neocarzinostat or cycdipepsynth or fom1 or bcpB or frbD or mitE or vlmB or prnB or CaiA or bacilysin or orf2\_PTase)

#### Legend:

core biosynthetic genes

additional biosynthetic genes

transport-related genes

regulatory genes

other genes

cluster extent as predicted by CASSIS

### Detailed annotation

### Homologous gene clusters

All hitsOidiodendron maius Zn unplaced genomic scaffold scaffold\_16, w... (100% of genes show similarity)Metarhizium anisopliae BRIP 53293 unplaced genomic scaffold Sc... (15% of genes show similarity)Metarhizium anisopliae ARSEF 549 scaffold\_7, whole genome ... (15% of genes show similarity)Trichophyton interdigitale H6 unplaced genomic scaffold superc... (15% of genes show similarity)Trichophyton interdigitale MR816 cont1.306, whole genome s... (15% of genes show similarity)Microsporum canis CBS 113480 supercont1.3 genomic scaffold, wh... (15% of genes show similarity)Metarhizium anisopliae BRIP 53284 unplaced genomic scaffold Sc... (15% of genes show similarity)Metarhizium robertsii strain ARSEF 2575 contig013, whole g... (15% of genes show similarity)Trichophyton rubrum CBS 202.88 unplaced genomic scaffold super... (15% of genes show similarity)Trichophyton rubrum CBS 288.86 unplaced genomic scaffold supe... (15% of genes show similarity)Download graphic

### scaffold\_17 - Cluster 40 - T1pks

### Predicted core structure

Rough prediction of core scaffold based on assumed PKS/NRPS colinearity; tailoring reactions not taken into account

### Prediction details

Monomers prediction:
:   N/A

### Gene cluster description

Download cluster GenBank file

scaffold\_17 - Gene Cluster 40. Type = t1pks. Location: 121852 - 170035 nt. Click on genes for more information.

Show pHMM detection rules used

t1pks: ((PKS\_KS & PKS\_AT) or (ene\_KS & PKS\_AT) or (mod\_KS & PKS\_AT) or (hyb\_KS & PKS\_AT) or (itr\_KS & PKS\_AT) or (tra\_KS & PKS\_AT))

#### Legend:

core biosynthetic genes

additional biosynthetic genes

transport-related genes

regulatory genes

other genes

cluster extent as predicted by CASSIS

### Detailed annotation

### Homologous gene clusters

All hitsOidiodendron maius Zn unplaced genomic scaffold scaffold\_17, w... (100% of genes show similarity)Aureobasidium pullulans var. pullulans EXF-150 unplaced genomi... (10% of genes show similarity)Aureobasidium pullulans var. melanogenum CBS 110374 unplaced g... (10% of genes show similarity)Phaeomoniella chlamydospora PC4\_UCR\_scaffold\_v01\_73, whole... (10% of genes show similarity)Stachybotrys chartarum IBT 7711 unplaced genomic scaffold scaf... (10% of genes show similarity)Stachybotrys chlorohalonata IBT 40285 unplaced genomic scaffol... (10% of genes show similarity)Stachybotrys chartarum IBT 40288 unplaced genomic scaffold sca... (10% of genes show similarity)Tolypocladium ophioglossoides CBS 100239 scaffold\_45, whol... (10% of genes show similarity)Zymoseptoria brevis strain Zb18110 contig4203, whole genom... (10% of genes show similarity)Aspergillus kawachii IFO 4308 DNA, contig: scaffold00002, who... (10% of genes show similarity)Download graphic

### scaffold\_17 - Cluster 41 - T1pks-nrps

### Predicted core structure

Rough prediction of core scaffold based on assumed PKS/NRPS colinearity; tailoring reactions not taken into account

### Prediction details

Monomers prediction:
:   N/A

### Database cross-links

Link to NORINE database query form  
Direct lookup in NORINE database in strict mode // relaxed mode

### Gene cluster description

Download cluster GenBank file

scaffold\_17 - Gene Cluster 41. Type = t1pks-nrps. Location: 250462 - 306229 nt. Click on genes for more information.

Show pHMM detection rules used

t1pks: ((PKS\_KS & PKS\_AT) or (ene\_KS & PKS\_AT) or (mod\_KS & PKS\_AT) or (hyb\_KS & PKS\_AT) or (itr\_KS & PKS\_AT) or (tra\_KS & PKS\_AT))  
nrps: ((Condensation & AMP-binding) or (Condensation & A-OX) or cluster(Condensation,AMP-binding))

#### Legend:

core biosynthetic genes

additional biosynthetic genes

transport-related genes

regulatory genes

other genes

cluster extent as predicted by CASSIS

### Detailed annotation

### Homologous gene clusters

All hitsOidiodendron maius Zn unplaced genomic scaffold scaffold\_17, w... (100% of genes show similarity)Acremonium chrysogenum ATCC 11550 scaffold33, whole genome... (23% of genes show similarity)Botryotinia fuckeliana BcDW1 unplaced genomic scaffold Scaffol... (17% of genes show similarity)Talaromyces islandicus genome assembly PIS, scaffold WF-38... (11% of genes show similarity)Mycosphaerella populorum SO2202 unplaced genomic scaffold SEPM... (11% of genes show similarity)Botryotinia fuckeliana T4 SuperContig\_51\_1 genomic supercontig. (11% of genes show similarity)Fusarium avenaceum strain Fa05001 Fave\_05001\_scaffold15.1,... (11% of genes show similarity)Pseudogymnoascus sp. VKM F-4513 (FW-928) contig\_2284, whol... (11% of genes show similarity)Fusarium graminearum chromosome 2, complete genome. (11% of genes show similarity)Fusarium graminearum PH-1 supercont3.5 genomic scaffold, whol... (11% of genes show similarity)Download graphic

### scaffold\_17 - Cluster 42 - Nrps

### Predicted core structure

Rough prediction of core scaffold based on assumed PKS/NRPS colinearity; tailoring reactions not taken into account

### Prediction details

Monomers prediction:
:   N/A

### Database cross-links

Link to NORINE database query form  
Direct lookup in NORINE database in strict mode // relaxed mode

### Gene cluster description

Download cluster GenBank file

scaffold\_17 - Gene Cluster 42. Type = nrps. Location: 395683 - 440566 nt. Click on genes for more information.

Show pHMM detection rules used

nrps: ((Condensation & AMP-binding) or (Condensation & A-OX) or cluster(Condensation,AMP-binding))

#### Legend:

core biosynthetic genes

additional biosynthetic genes

transport-related genes

regulatory genes

other genes

cluster extent as predicted by CASSIS

### Detailed annotation

### Homologous gene clusters

All hitsOidiodendron maius Zn unplaced genomic scaffold scaffold\_17, w... (93% of genes show similarity)Aspergillus clavatus NRRL 1 1099423829799 genomic scaffold, wh... (43% of genes show similarity)Madurella mycetomatis strain mm55 MMYC01\_0026, whole genom... (43% of genes show similarity)Trichophyton interdigitale MR816 cont1.203, whole genome s... (43% of genes show similarity)Trichophyton equinum CBS 127.97 supercont1.18 genomic scaffold... (43% of genes show similarity)Trichophyton rubrum CBS 288.86 unplaced genomic scaffold super... (43% of genes show similarity)Trichophyton rubrum CBS 100081 chromosome Unknown supercont1.2... (43% of genes show similarity)Trichophyton rubrum MR1448 unplaced genomic scaffold supercont... (43% of genes show similarity)Trichophyton rubrum MR850 unplaced genomic scaffold supercont1... (43% of genes show similarity)Trichophyton rubrum CBS 202.88 unplaced genomic scaffold supe... (43% of genes show similarity)Download graphic

### Homologous known gene clusters

All hitsAspirochlorine biosynthetic gene cluster (18% of genes show similarity)Download graphic

### scaffold\_18 - Cluster 43 - Siderophore

### Gene cluster description

Download cluster GenBank file

scaffold\_18 - Gene Cluster 43. Type = siderophore. Location: 589671 - 600870 nt. Click on genes for more information.

Show pHMM detection rules used

siderophore: (IucA\_IucC)

#### Legend:

core biosynthetic genes

additional biosynthetic genes

transport-related genes

regulatory genes

other genes

cluster extent as predicted by CASSIS

### Homologous gene clusters

All hitsOidiodendron maius Zn unplaced genomic scaffold scaffold\_18, w... (26% of genes show similarity)Download graphic

### scaffold\_20 - Cluster 44 - T1pks

### Predicted core structure

Rough prediction of core scaffold based on assumed PKS/NRPS colinearity; tailoring reactions not taken into account

### Prediction details

Monomers prediction:
:   N/A

### Gene cluster description

Download cluster GenBank file

scaffold\_20 - Gene Cluster 44. Type = t1pks. Location: 34834 - 81611 nt. Click on genes for more information.

Show pHMM detection rules used

t1pks: ((PKS\_KS & PKS\_AT) or (ene\_KS & PKS\_AT) or (mod\_KS & PKS\_AT) or (hyb\_KS & PKS\_AT) or (itr\_KS & PKS\_AT) or (tra\_KS & PKS\_AT))

#### Legend:

core biosynthetic genes

additional biosynthetic genes

transport-related genes

regulatory genes

other genes

cluster extent as predicted by CASSIS

### Detailed annotation

### Homologous gene clusters

All hitsOidiodendron maius Zn unplaced genomic scaffold scaffold\_20, w... (100% of genes show similarity)Glomerella graminicola M1.001 genomic scaffold supercont1.42, ... (33% of genes show similarity)Rasamsonia emersonii CBS 393.64 ctg1118621760004, whole ge... (20% of genes show similarity)Oidiodendron maius Zn unplaced genomic scaffold scaffold\_1, wh... (20% of genes show similarity)Magnaporthe oryzae 70-15 chromosome 5, whole genome shotgun se... (26% of genes show similarity)Colletotrichum orbiculare MAFF 240422 unplaced genomic scaffol... (20% of genes show similarity)Magnaporthe oryzae Y34 unplaced genomic scaffold Y34\_scaffold0... (26% of genes show similarity)Colletotrichum fioriniae PJ7 Scaffold\_183.1, whole genome ... (26% of genes show similarity)Colletotrichum gloeosporioides Nara gc5 unplaced genomic scaff... (20% of genes show similarity)Aspergillus nomius NRRL 13137 contig00003, whole genome s... (13% of genes show similarity)Download graphic

### scaffold\_20 - Cluster 45 - T1pks

### Predicted core structure

Rough prediction of core scaffold based on assumed PKS/NRPS colinearity; tailoring reactions not taken into account

### Prediction details

Monomers prediction:
:   N/A

### Gene cluster description

Download cluster GenBank file

scaffold\_20 - Gene Cluster 45. Type = t1pks. Location: 498019 - 546308 nt. Click on genes for more information.

Show pHMM detection rules used

t1pks: ((PKS\_KS & PKS\_AT) or (ene\_KS & PKS\_AT) or (mod\_KS & PKS\_AT) or (hyb\_KS & PKS\_AT) or (itr\_KS & PKS\_AT) or (tra\_KS & PKS\_AT))

#### Legend:

core biosynthetic genes

additional biosynthetic genes

transport-related genes

regulatory genes

other genes

cluster extent as predicted by CASSIS

### Detailed annotation

### Homologous gene clusters

All hitsOidiodendron maius Zn unplaced genomic scaffold scaffold\_20, w... (100% of genes show similarity)Oidiodendron maius Zn unplaced genomic scaffold scaffold\_9, wh... (13% of genes show similarity)Rasamsonia emersonii CBS 393.64 ctg1118621759978, whole ge... (13% of genes show similarity)Talaromyces islandicus genome assembly PIS, scaffold WF-38... (13% of genes show similarity)Gaeumannomyces graminis var. tritici R3-111a-1 unplaced genomi... (13% of genes show similarity)Diaporthe ampelina DA\_912\_scaffold\_v01\_11, whole genome sh... (13% of genes show similarity)Arthroderma gypseum CBS 118893 supercont1.6 genomic scaffold, ... (13% of genes show similarity)Neofusicoccum parvum UCRNP2 chromosome Unknown NP2\_03\_scaffold... (13% of genes show similarity)Aspergillus terreus NIH2624 scaffold\_10 genomic scaffold, whol... (20% of genes show similarity)Penicillium marneffei ATCC 18224 scf\_1105668340758 genomic sc... (13% of genes show similarity)Download graphic

### scaffold\_20 - Cluster 46 - T1pks-nrps

### Predicted core structure

Rough prediction of core scaffold based on assumed PKS/NRPS colinearity; tailoring reactions not taken into account

### Prediction details

Monomers prediction:
:   N/A

### Database cross-links

Link to NORINE database query form  
Direct lookup in NORINE database in strict mode // relaxed mode

### Gene cluster description

Download cluster GenBank file

scaffold\_20 - Gene Cluster 46. Type = t1pks-nrps. Location: 795399 - 850123 nt. Click on genes for more information.

Show pHMM detection rules used

t1pks: ((PKS\_KS & PKS\_AT) or (ene\_KS & PKS\_AT) or (mod\_KS & PKS\_AT) or (hyb\_KS & PKS\_AT) or (itr\_KS & PKS\_AT) or (tra\_KS & PKS\_AT))  
nrps: ((Condensation & AMP-binding) or (Condensation & A-OX) or cluster(Condensation,AMP-binding))

#### Legend:

core biosynthetic genes

additional biosynthetic genes

transport-related genes

regulatory genes

other genes

cluster extent as predicted by CASSIS

### Detailed annotation

### Homologous gene clusters

All hitsOidiodendron maius Zn unplaced genomic scaffold scaffold\_20, w... (100% of genes show similarity)Eutypa lata UCREL1 unplaced genomic scaffold EL1\_03\_scaffold\_2... (20% of genes show similarity)Talaromyces stipitatus ATCC 10500 scf\_1105507295511 genomic sc... (13% of genes show similarity)Acremonium chrysogenum ATCC 11550 scaffold9, whole genome ... (13% of genes show similarity)Metarhizium guizhouense ARSEF 977 scaffold\_25, whole genom... (13% of genes show similarity)Metarhizium acridum CQMa 102 unplaced genomic scaffold Scf\_155... (13% of genes show similarity)Metarhizium anisopliae ARSEF 549 scaffold\_11, whole genome... (13% of genes show similarity)Metarhizium anisopliae strain E6 contig00039, whole genome... (13% of genes show similarity)Metarhizium anisopliae BRIP 53293 unplaced genomic scaffold Sc... (13% of genes show similarity)Metarhizium anisopliae BRIP 53284 unplaced genomic scaffold S... (13% of genes show similarity)Download graphic

### scaffold\_21 - Cluster 47 - T1pks

### Predicted core structure

Rough prediction of core scaffold based on assumed PKS/NRPS colinearity; tailoring reactions not taken into account

### Prediction details

Monomers prediction:
:   (pk) + (mal)

jgi.p\_Oidma1\_172777
:   PKS signature: mal
:   Minowa: mal
:   consensus: mal

jgi.p\_Oidma1\_106798
:   PKS signature: mal
:   Minowa: mmal
:   consensus: pk

### Gene cluster description

Download cluster GenBank file

scaffold\_21 - Gene Cluster 47. Type = t1pks. Location: 683224 - 738236 nt. Click on genes for more information.

Show pHMM detection rules used

t1pks: ((PKS\_KS & PKS\_AT) or (ene\_KS & PKS\_AT) or (mod\_KS & PKS\_AT) or (hyb\_KS & PKS\_AT) or (itr\_KS & PKS\_AT) or (tra\_KS & PKS\_AT))

#### Legend:

core biosynthetic genes

additional biosynthetic genes

transport-related genes

regulatory genes

other genes

cluster extent as predicted by CASSIS

### Detailed annotation

### Homologous gene clusters

All hitsOidiodendron maius Zn unplaced genomic scaffold scaffold\_21, w... (100% of genes show similarity)Diaporthe ampelina DA\_912\_scaffold\_v01\_427, whole genome s... (23% of genes show similarity)Setosphaeria turcica Et28A unplaced genomic scaffold SETTUscaf... (29% of genes show similarity)Colletotrichum orbiculare MAFF 240422 unplaced genomic scaffol... (23% of genes show similarity)Metarhizium anisopliae BRIP 53293 unplaced genomic scaffold Sc... (29% of genes show similarity)Penicillium marneffei ATCC 18224 scf\_1105668340984 genomic sca... (23% of genes show similarity)Exophiala xenobiotica strain CBS 118157 unplaced genomic scaff... (23% of genes show similarity)Exophiala oligosperma strain CBS 72588 unplaced genomic scaffo... (23% of genes show similarity)Pseudogymnoascus sp. VKM F-3775 contig\_4145, whole genome ... (23% of genes show similarity)Penicillium chrysogenum Wisconsin 54-1255 complete genome, co... (17% of genes show similarity)Download graphic

### Homologous known gene clusters

All hitsZearalenone biosynthetic gene cluster (18% of genes show similarity)Hypothemycin biosynthetic gene cluster (27% of genes show similarity)Hypothemycin biosynthetic gene cluster (27% of genes show similarity)Dehydrocurvularin biosynthetic gene cluster (18% of genes show similarity)Lasiodiplodin biosynthetic gene cluster (18% of genes show similarity)Download graphic

### scaffold\_22 - Cluster 48 - T1pks-nrps

### Predicted core structure

Rough prediction of core scaffold based on assumed PKS/NRPS colinearity; tailoring reactions not taken into account

### Prediction details

Monomers prediction:
:   N/A

### Database cross-links

Link to NORINE database query form  
Direct lookup in NORINE database in strict mode // relaxed mode

### Gene cluster description

Download cluster GenBank file

scaffold\_22 - Gene Cluster 48. Type = t1pks-nrps. Location: 8543 - 60967 nt. Click on genes for more information.

Show pHMM detection rules used

t1pks: ((PKS\_KS & PKS\_AT) or (ene\_KS & PKS\_AT) or (mod\_KS & PKS\_AT) or (hyb\_KS & PKS\_AT) or (itr\_KS & PKS\_AT) or (tra\_KS & PKS\_AT))  
nrps: ((Condensation & AMP-binding) or (Condensation & A-OX) or cluster(Condensation,AMP-binding))

#### Legend:

core biosynthetic genes

additional biosynthetic genes

transport-related genes

regulatory genes

other genes

cluster extent as predicted by CASSIS

### Detailed annotation

### Homologous gene clusters

All hitsOidiodendron maius Zn unplaced genomic scaffold scaffold\_22, w... (100% of genes show similarity)Myceliophthora thermophila ATCC 42464 chromosome 3, complete s... (52% of genes show similarity)Metarhizium robertsii ARSEF 23 MAA\_Scf\_9, whole genome sho... (23% of genes show similarity)Metarhizium guizhouense ARSEF 977 scaffold\_76, whole genom... (23% of genes show similarity)Metarhizium majus ARSEF 297 Scaffold\_167, whole genome sho... (23% of genes show similarity)Parastagonospora nodorum SN15 scaffold\_1, whole genome shotgun... (23% of genes show similarity)Neosartorya udagawae DNA, contig:Aud0008, strain: IFM 4697... (23% of genes show similarity)Beauveria bassiana ARSEF 2860 unplaced genomic scaffold BBA\_S0... (23% of genes show similarity)Metarhizium robertsii strain ARSEF 2575 contig062, whole g... (23% of genes show similarity)Colletotrichum orbiculare MAFF 240422 unplaced genomic scaffo... (23% of genes show similarity)Download graphic

### scaffold\_22 - Cluster 49 - T1pks

### Predicted core structure

Rough prediction of core scaffold based on assumed PKS/NRPS colinearity; tailoring reactions not taken into account

### Prediction details

Monomers prediction:
:   N/A

### Gene cluster description

Download cluster GenBank file

scaffold\_22 - Gene Cluster 49. Type = t1pks. Location: 208990 - 292176 nt. Click on genes for more information.

Show pHMM detection rules used

t1pks: ((PKS\_KS & PKS\_AT) or (ene\_KS & PKS\_AT) or (mod\_KS & PKS\_AT) or (hyb\_KS & PKS\_AT) or (itr\_KS & PKS\_AT) or (tra\_KS & PKS\_AT))

#### Legend:

core biosynthetic genes

additional biosynthetic genes

transport-related genes

regulatory genes

other genes

cluster extent as predicted by CASSIS

### Detailed annotation

### Homologous gene clusters

All hitsOidiodendron maius Zn unplaced genomic scaffold scaffold\_22, w... (100% of genes show similarity)Pseudogymnoascus sp. VKM F-103 contig\_31, whole genome sho... (36% of genes show similarity)Pseudogymnoascus sp. VKM F-4519 (FW-2642) contig\_228, whol... (32% of genes show similarity)Pseudogymnoascus sp. VKM F-3808 contig\_584, whole genome s... (28% of genes show similarity)Pseudogymnoascus sp. VKM F-3557 contig\_596, whole genome s... (28% of genes show similarity)Fusarium oxysporum f. sp. vasinfectum 25433 unplaced genomic s... (20% of genes show similarity)Fusarium fujikuroi strain KSU 3368 seq\_1893, whole genome ... (20% of genes show similarity)Fusarium oxysporum Fo5176 contig02053, whole genome shotgu... (20% of genes show similarity)Fusarium oxysporum f. sp. lycopersici MN25 unplaced genomic sc... (28% of genes show similarity)Fusarium oxysporum f. sp. lycopersici 4287 supercont2.12 geno... (28% of genes show similarity)Download graphic

### Homologous known gene clusters

All hitsCitrinin biosynthetic gene cluster (12% of genes show similarity)Azaphilone biosynthetic gene cluster (25% of genes show similarity)Azanigerone biosynthetic gene cluster (18% of genes show similarity)Stipitatic acid biosynthetic gene cluster (12% of genes show similarity)Asperfuranone biosynthetic gene cluster (12% of genes show similarity)Download graphic

### scaffold\_23 - Cluster 50 - T1pks-nrps

### Predicted core structure

Rough prediction of core scaffold based on assumed PKS/NRPS colinearity; tailoring reactions not taken into account

### Prediction details

Monomers prediction:
:   N/A

### Database cross-links

Link to NORINE database query form  
Direct lookup in NORINE database in strict mode // relaxed mode

### Gene cluster description

Download cluster GenBank file

scaffold\_23 - Gene Cluster 50. Type = t1pks-nrps. Location: 147253 - 213241 nt. Click on genes for more information.

Show pHMM detection rules used

t1pks: ((PKS\_KS & PKS\_AT) or (ene\_KS & PKS\_AT) or (mod\_KS & PKS\_AT) or (hyb\_KS & PKS\_AT) or (itr\_KS & PKS\_AT) or (tra\_KS & PKS\_AT))  
nrps: ((Condensation & AMP-binding) or (Condensation & A-OX) or cluster(Condensation,AMP-binding))

#### Legend:

core biosynthetic genes

additional biosynthetic genes

transport-related genes

regulatory genes

other genes

cluster extent as predicted by CASSIS

### Detailed annotation

### Homologous gene clusters

All hitsOidiodendron maius Zn unplaced genomic scaffold scaffold\_23, w... (100% of genes show similarity)Stachybotrys chlorohalonata IBT 40285 unplaced genomic scaffol... (18% of genes show similarity)Hirsutella minnesotensis 3608 unplaced genomic scaffold MNSD\_S... (18% of genes show similarity)Aspergillus niger contig An11c0220, genomic contig. (13% of genes show similarity)Aspergillus niger ATCC 1015, whole genome shotgun sequence. (13% of genes show similarity)Aspergillus kawachii IFO 4308 DNA, contig: scaffold00054, whol... (13% of genes show similarity)Mycosphaerella graminicola IPO323 chromosome 10, whole genome ... (13% of genes show similarity)Pseudocercospora fijiensis CIRAD86 unplaced genomic scaffold M... (13% of genes show similarity)Bipolaris victoriae FI3 unplaced genomic scaffold scaffold\_123... (18% of genes show similarity)Fusarium verticillioides 7600 chromosome 9 supercont3.16, who... (13% of genes show similarity)Download graphic

### Homologous known gene clusters

All hitsAflatrem biosynthetic gene cluster (11% of genes show similarity)Download graphic

### scaffold\_26 - Cluster 51 - Indole-t1pks

### Predicted core structure

Rough prediction of core scaffold based on assumed PKS/NRPS colinearity; tailoring reactions not taken into account

### Prediction details

Monomers prediction:
:   (mal)

jgi.p\_Oidma1\_173223
:   PKS signature: mal
:   Minowa: mal
:   consensus: mal

### Gene cluster description

Download cluster GenBank file

scaffold\_26 - Gene Cluster 51. Type = indole-t1pks. Location: 109140 - 176694 nt. Click on genes for more information.

Show pHMM detection rules used

indole: (indsynth or dmat or indole\_PTase)  
t1pks: ((PKS\_KS & PKS\_AT) or (ene\_KS & PKS\_AT) or (mod\_KS & PKS\_AT) or (hyb\_KS & PKS\_AT) or (itr\_KS & PKS\_AT) or (tra\_KS & PKS\_AT))

#### Legend:

core biosynthetic genes

additional biosynthetic genes

transport-related genes

regulatory genes

other genes

cluster extent as predicted by CASSIS

### Detailed annotation

### Homologous gene clusters

All hitsOidiodendron maius Zn unplaced genomic scaffold scaffold\_26, w... (100% of genes show similarity)Penicillium brasilianum genome assembly Pbras\_Allpaths-LG,... (44% of genes show similarity)Penicillium aethiopicum strain IBT 5753 viridicatumtoxin biosy... (44% of genes show similarity)Aspergillus ustus strain 3.3904 AUSC\_33, whole genome shot... (32% of genes show similarity)Pseudogymnoascus sp. VKM F-3775 contig\_1118, whole genome ... (32% of genes show similarity)Metarhizium majus ARSEF 297 Scaffold\_31, whole genome shot... (28% of genes show similarity)Metarhizium acridum CQMa 102 unplaced genomic scaffold Scf\_026... (28% of genes show similarity)Metarhizium album ARSEF 1941 scaffold\_10, whole genome sho... (28% of genes show similarity)Aspergillus fumigatus A1163 scf\_000008 genomic scaffold, whole... (28% of genes show similarity)Aspergillus fumigatus Z5 chromosome Unknown scaffold\_14, whol... (28% of genes show similarity)Download graphic

### Homologous known gene clusters

All hitsViridicatumtoxin biosynthetic gene cluster (40% of genes show similarity)TAN-1612 biosynthetic gene cluster (18% of genes show similarity)Neosartoricin biosynthetic gene cluster (13% of genes show similarity)Asperthecin biosynthetic gene cluster (13% of genes show similarity)Download graphic

### scaffold\_28 - Cluster 52 - T1pks

### Predicted core structure

Rough prediction of core scaffold based on assumed PKS/NRPS colinearity; tailoring reactions not taken into account

### Prediction details

Monomers prediction:
:   N/A

### Gene cluster description

Download cluster GenBank file

scaffold\_28 - Gene Cluster 52. Type = t1pks. Location: 83384 - 138964 nt. Click on genes for more information.

Show pHMM detection rules used

t1pks: ((PKS\_KS & PKS\_AT) or (ene\_KS & PKS\_AT) or (mod\_KS & PKS\_AT) or (hyb\_KS & PKS\_AT) or (itr\_KS & PKS\_AT) or (tra\_KS & PKS\_AT))

#### Legend:

core biosynthetic genes

additional biosynthetic genes

transport-related genes

regulatory genes

other genes

cluster extent as predicted by CASSIS

### Detailed annotation

### Homologous gene clusters

All hitsOidiodendron maius Zn unplaced genomic scaffold scaffold\_28, w... (100% of genes show similarity)Chaetomium globosum CBS 148.51 scaffold\_5 genomic scaffold, wh... (33% of genes show similarity)Aspergillus nidulans FGSC A4 chromosome IV ANcontig1.118, ... (25% of genes show similarity)Setosphaeria turcica Et28A unplaced genomic scaffold SETTUscaf... (25% of genes show similarity)Sorangium cellulosum So0157-2, complete genome. (33% of genes show similarity)Oidiodendron maius Zn unplaced genomic scaffold scaffold\_17, w... (16% of genes show similarity)Cochliobolus sativus ND90Pr unplaced genomic scaffold COCSAsca... (16% of genes show similarity)Colletotrichum sublineola strain TX430BB Scaffolds0713.1, ... (25% of genes show similarity)Penicillium marneffei ATCC 18224 scf\_1105668340984 genomic sca... (25% of genes show similarity)Talaromyces marneffei PM1 scf057, whole genome shotgun se... (25% of genes show similarity)Download graphic

### scaffold\_28 - Cluster 53 - T1pks-nrps

### Predicted core structure

Rough prediction of core scaffold based on assumed PKS/NRPS colinearity; tailoring reactions not taken into account

### Prediction details

Monomers prediction:
:   N/A

### Database cross-links

Link to NORINE database query form  
Direct lookup in NORINE database in strict mode // relaxed mode

### Gene cluster description

Download cluster GenBank file

scaffold\_28 - Gene Cluster 53. Type = t1pks-nrps. Location: 177158 - 232041 nt. Click on genes for more information.

Show pHMM detection rules used

t1pks: ((PKS\_KS & PKS\_AT) or (ene\_KS & PKS\_AT) or (mod\_KS & PKS\_AT) or (hyb\_KS & PKS\_AT) or (itr\_KS & PKS\_AT) or (tra\_KS & PKS\_AT))  
nrps: ((Condensation & AMP-binding) or (Condensation & A-OX) or cluster(Condensation,AMP-binding))

#### Legend:

core biosynthetic genes

additional biosynthetic genes

transport-related genes

regulatory genes

other genes

cluster extent as predicted by CASSIS

### Detailed annotation

### Homologous gene clusters

All hitsOidiodendron maius Zn unplaced genomic scaffold scaffold\_28, w... (100% of genes show similarity)Endocarpon pusillum Z07020 unplaced genomic scaffold scaffold6... (20% of genes show similarity)Eutypa lata UCREL1 unplaced genomic scaffold EL1\_03\_scaffold\_2... (20% of genes show similarity)Pestalotiopsis fici W106-1 unplaced genomic scaffold PFICI\_Scf... (20% of genes show similarity)Stachybotrys chartarum IBT 7711 unplaced genomic scaffold scaf... (20% of genes show similarity)Stachybotrys chartarum IBT 40288 unplaced genomic scaffold sca... (20% of genes show similarity)Stachybotrys chartarum IBT 40293 unplaced genomic scaffold sca... (20% of genes show similarity)Stachybotrys chlorohalonata IBT 40285 unplaced genomic scaffol... (20% of genes show similarity)Chaetomium globosum CBS 148.51 scaffold\_4 genomic scaffold, wh... (20% of genes show similarity)Madurella mycetomatis strain mm55 MMYC01\_0270, whole geno... (20% of genes show similarity)Download graphic

### scaffold\_33 - Cluster 54 - T1pks

### Predicted core structure

Rough prediction of core scaffold based on assumed PKS/NRPS colinearity; tailoring reactions not taken into account

### Prediction details

Monomers prediction:
:   (mal)

jgi.p\_Oidma1\_138681
:   PKS signature: mal
:   Minowa: mal
:   consensus: mal

### Gene cluster description

Download cluster GenBank file

scaffold\_33 - Gene Cluster 54. Type = t1pks. Location: 59164 - 106713 nt. Click on genes for more information.

Show pHMM detection rules used

t1pks: ((PKS\_KS & PKS\_AT) or (ene\_KS & PKS\_AT) or (mod\_KS & PKS\_AT) or (hyb\_KS & PKS\_AT) or (itr\_KS & PKS\_AT) or (tra\_KS & PKS\_AT))

#### Legend:

core biosynthetic genes

additional biosynthetic genes

transport-related genes

regulatory genes

other genes

cluster extent as predicted by CASSIS

### Detailed annotation

### Homologous gene clusters

All hitsOidiodendron maius Zn unplaced genomic scaffold scaffold\_33, w... (100% of genes show similarity)Scedosporium apiospermum strain IHEM 14462 SEQ\_SAPIO\_0094,... (26% of genes show similarity)Stachybotrys chartarum IBT 40293 unplaced genomic scaffold sca... (26% of genes show similarity)Stachybotrys chlorohalonata IBT 40285 unplaced genomic scaffol... (26% of genes show similarity)Stachybotrys chartarum IBT 7711 unplaced genomic scaffold scaf... (26% of genes show similarity)Hirsutella minnesotensis 3608 unplaced genomic scaffold MNSD\_S... (20% of genes show similarity)Madurella mycetomatis strain mm55 MMYC01\_0453, whole genom... (20% of genes show similarity)Stachybotrys chartarum IBT 40288 unplaced genomic scaffold sca... (20% of genes show similarity)Setosphaeria turcica Et28A unplaced genomic scaffold SETTUscaf... (26% of genes show similarity)Pyrenophora teres f. teres 0-1 unplaced genomic scaffold scaf... (26% of genes show similarity)Download graphic

### scaffold\_34 - Cluster 55 - T1pks-nrps

### Predicted core structure

Rough prediction of core scaffold based on assumed PKS/NRPS colinearity; tailoring reactions not taken into account

### Prediction details

Monomers prediction:
:   N/A

### Database cross-links

Link to NORINE database query form  
Direct lookup in NORINE database in strict mode // relaxed mode

### Gene cluster description

Download cluster GenBank file

scaffold\_34 - Gene Cluster 55. Type = t1pks-nrps. Location: 25376 - 108672 nt. Click on genes for more information.

Show pHMM detection rules used

t1pks: ((PKS\_KS & PKS\_AT) or (ene\_KS & PKS\_AT) or (mod\_KS & PKS\_AT) or (hyb\_KS & PKS\_AT) or (itr\_KS & PKS\_AT) or (tra\_KS & PKS\_AT))  
nrps: ((Condensation & AMP-binding) or (Condensation & A-OX) or cluster(Condensation,AMP-binding))

#### Legend:

core biosynthetic genes

additional biosynthetic genes

transport-related genes

regulatory genes

other genes

cluster extent as predicted by CASSIS

### Detailed annotation

### Homologous gene clusters

All hitsOidiodendron maius Zn unplaced genomic scaffold scaffold\_34, w... (100% of genes show similarity)Pseudogymnoascus sp. VKM F-4515 (FW-2607) contig\_1873, who... (48% of genes show similarity)Pseudogymnoascus sp. VKM F-4517 (FW-2822) contig\_1345, who... (41% of genes show similarity)Pseudogymnoascus sp. VKM F-4520 (FW-2644) contig\_4271, who... (34% of genes show similarity)Pseudogymnoascus sp. VKM F-4518 (FW-2643) contig\_3667, who... (34% of genes show similarity)Aspergillus fumigatus Af293 chromosome 4 afu1\_96, whole ge... (37% of genes show similarity)Aspergillus fumigatus Z5 chromosome Unknown scaffold\_12, whole... (37% of genes show similarity)Verruconis gallopava strain CBS 43764 unplaced genomic scaffol... (37% of genes show similarity)Aspergillus fumigatus var. RP-2014 strain niveus afvn\_518,... (34% of genes show similarity)Aspergillus terreus NIH2624 scaffold\_12 genomic scaffold, who... (34% of genes show similarity)Download graphic

### Homologous known gene clusters

All hitsTrypacidin biosynthetic gene cluster (71% of genes show similarity)Pestheic acid biosynthetic gene cluster (50% of genes show similarity)Emericellin biosynthetic gene cluster (28% of genes show similarity)Endocrocin biosynthetic gene cluster (14% of genes show similarity)TAN-1612 biosynthetic gene cluster (14% of genes show similarity)Asperthecin biosynthetic gene cluster (14% of genes show similarity)Equisetin biosynthetic gene cluster (14% of genes show similarity)Neosartoricin biosynthetic gene cluster (14% of genes show similarity)Viridicatumtoxin biosynthetic gene cluster (14% of genes show similarity)Notoamide / stephacidin biosynthetic gene cluster (21% of genes show similarity)Download graphic

### scaffold\_35 - Cluster 56 - T1pks-nrps

### Predicted core structure

Rough prediction of core scaffold based on assumed PKS/NRPS colinearity; tailoring reactions not taken into account

### Prediction details

Monomers prediction:
:   N/A

### Database cross-links

Link to NORINE database query form  
Direct lookup in NORINE database in strict mode // relaxed mode

### Gene cluster description

Download cluster GenBank file

scaffold\_35 - Gene Cluster 56. Type = t1pks-nrps. Location: 7178 - 101950 nt. Click on genes for more information.

Show pHMM detection rules used

t1pks: ((PKS\_KS & PKS\_AT) or (ene\_KS & PKS\_AT) or (mod\_KS & PKS\_AT) or (hyb\_KS & PKS\_AT) or (itr\_KS & PKS\_AT) or (tra\_KS & PKS\_AT))  
nrps: ((Condensation & AMP-binding) or (Condensation & A-OX) or cluster(Condensation,AMP-binding))

#### Legend:

core biosynthetic genes

additional biosynthetic genes

transport-related genes

regulatory genes

other genes

cluster extent as predicted by CASSIS

### Detailed annotation

### Homologous gene clusters

All hitsOidiodendron maius Zn unplaced genomic scaffold scaffold\_35, w... (100% of genes show similarity)Endocarpon pusillum Z07020 unplaced genomic scaffold scaffold3... (23% of genes show similarity)Pyrenophora tritici-repentis Pt-1C-BFP supercont1.10 genomic s... (15% of genes show similarity)Aspergillus nomius NRRL 13137 contig00086, whole genome sh... (15% of genes show similarity)Aspergillus flavus NRRL3357 scf\_1106287692089 genomic scaffold... (15% of genes show similarity)Aspergillus flavus NRRL3357 scf\_1106286418500 genomic scaffold... (11% of genes show similarity)Aspergillus oryzae RIB40 DNA, SC001. (11% of genes show similarity)Sordaria macrospora k-hell, WGS project CABT00000000 data,... (11% of genes show similarity)Magnaporthe oryzae 70-15 chromosome 1, whole genome shotgun se... (11% of genes show similarity)Pestalotiopsis fici W106-1 unplaced genomic scaffold PFICI\_Sc... (11% of genes show similarity)Download graphic

### scaffold\_35 - Cluster 57 - Other

### Predicted core structure

Rough prediction of core scaffold based on assumed PKS/NRPS colinearity; tailoring reactions not taken into account

### Prediction details

Monomers prediction:
:   N/A

### Gene cluster description

Download cluster GenBank file

scaffold\_35 - Gene Cluster 57. Type = other. Location: 110138 - 150436 nt. Click on genes for more information.

Show pHMM detection rules used

other: ((PP-binding & AMP-binding) or (PP-binding & A-OX) or (NAD\_binding\_4 & A-OX) or (NAD\_binding\_4 & AMP-binding) or LmbU or Neocarzinostat or cycdipepsynth or fom1 or bcpB or frbD or mitE or vlmB or prnB or CaiA or bacilysin or orf2\_PTase)

#### Legend:

core biosynthetic genes

additional biosynthetic genes

transport-related genes

regulatory genes

other genes

cluster extent as predicted by CASSIS

### Detailed annotation

### Homologous gene clusters

All hitsOidiodendron maius Zn unplaced genomic scaffold scaffold\_35, w... (100% of genes show similarity)Cladophialophora immunda strain CBS 83496 unplaced genomic sca... (15% of genes show similarity)Burkholderia rhizoxinica HKI 454 plasmid pBRH01, complete sequ... (30% of genes show similarity)Bradyrhizobium sp. ORS 285, WGS project CAFH00000000 data,... (23% of genes show similarity)Nocardia vulneris strain W9851 contig00009, whole genome s... (23% of genes show similarity)Nocardia brasiliensis ATCC 700358, complete genome. (23% of genes show similarity)Nocardia brasiliensis NBRC 14402 DNA, contig: NBRGN061, wh... (23% of genes show similarity)Rhodococcus aetherivorans strain IcdP1, complete genome. (23% of genes show similarity)Pseudomonas sp. WCS358 WCS358-Sc1, whole genome shotgun se... (23% of genes show similarity)Pseudomonas sp. 655 contig\_13, whole genome shotgun seque... (23% of genes show similarity)Download graphic

### scaffold\_38 - Cluster 58 - T1pks-nrps

### Predicted core structure

Rough prediction of core scaffold based on assumed PKS/NRPS colinearity; tailoring reactions not taken into account

### Prediction details

Monomers prediction:
:   N/A

### Database cross-links

Link to NORINE database query form  
Direct lookup in NORINE database in strict mode // relaxed mode

### Gene cluster description

Download cluster GenBank file

scaffold\_38 - Gene Cluster 58. Type = t1pks-nrps. Location: 25729 - 114735 nt. Click on genes for more information.

Show pHMM detection rules used

t1pks: ((PKS\_KS & PKS\_AT) or (ene\_KS & PKS\_AT) or (mod\_KS & PKS\_AT) or (hyb\_KS & PKS\_AT) or (itr\_KS & PKS\_AT) or (tra\_KS & PKS\_AT))  
nrps: ((Condensation & AMP-binding) or (Condensation & A-OX) or cluster(Condensation,AMP-binding))

#### Legend:

core biosynthetic genes

additional biosynthetic genes

transport-related genes

regulatory genes

other genes

cluster extent as predicted by CASSIS

### Detailed annotation

### Homologous gene clusters

All hitsOidiodendron maius Zn unplaced genomic scaffold scaffold\_38, w... (100% of genes show similarity)Tolypocladium ophioglossoides CBS 100239 scaffold\_45, whol... (27% of genes show similarity)Penicillium oxalicum 114-2 unplaced genomic scaffold scaffold\_... (22% of genes show similarity)Fungal sp. No.11243 DNA, scaffold: sccafold0004, whole genome ... (22% of genes show similarity)Colletotrichum sublineola strain TX430BB Scaffolds0010.1, ... (16% of genes show similarity)Glomerella graminicola M1.001 genomic scaffold supercont1.87, ... (16% of genes show similarity)Trichoderma atroviride IMI 206040 TRIATcontig\_22, whole ge... (16% of genes show similarity)Talaromyces marneffei PM1 scf061, whole genome shotgun seq... (22% of genes show similarity)Penicillium marneffei ATCC 18224 scf\_1105668340970 genomic sca... (22% of genes show similarity)Glarea lozoyensis ATCC 20868 chromosome Unknown GLAREA13, who... (16% of genes show similarity)Download graphic

### scaffold\_54 - Cluster 59 - T1pks

### Predicted core structure

Rough prediction of core scaffold based on assumed PKS/NRPS colinearity; tailoring reactions not taken into account

### Prediction details

Monomers prediction:
:   (mal)

jgi.p\_Oidma1\_62490
:   PKS signature: mal
:   Minowa: mal
:   consensus: mal

### Gene cluster description

Download cluster GenBank file

scaffold\_54 - Gene Cluster 59. Type = t1pks. Location: 1 - 30982 nt. Click on genes for more information.

Show pHMM detection rules used

t1pks: ((PKS\_KS & PKS\_AT) or (ene\_KS & PKS\_AT) or (mod\_KS & PKS\_AT) or (hyb\_KS & PKS\_AT) or (itr\_KS & PKS\_AT) or (tra\_KS & PKS\_AT))

#### Legend:

core biosynthetic genes

additional biosynthetic genes

transport-related genes

regulatory genes

other genes

cluster extent as predicted by CASSIS

### Detailed annotation

### Homologous gene clusters

All hitsOidiodendron maius Zn unplaced genomic scaffold scaffold\_54, w... (100% of genes show similarity)Neosartorya udagawae DNA, contig:Aud0008, strain: IFM 4697... (22% of genes show similarity)Podospora anserina genomic DNA, chromosome 2. (22% of genes show similarity)Podospora anserina S mat+ genomic DNA chromosome 2, superconti... (22% of genes show similarity)Aspergillus ruber CBS 135680 unplaced genomic scaffold scaffol... (22% of genes show similarity)Oidiodendron maius Zn unplaced genomic scaffold scaffold\_14, w... (22% of genes show similarity)Cystobacter fuscus cystothiazole A gene cluster, complete sequ... (33% of genes show similarity)Penicillium brasilianum genome assembly Pbras\_Allpaths-LG,... (22% of genes show similarity)Aspergillus clavatus NRRL 1 1099423829792 genomic scaffold, wh... (22% of genes show similarity)Metarhizium guizhouense ARSEF 977 scaffold\_42, whole geno... (22% of genes show similarity)Download graphic

|  |  |  |  |
| --- | --- | --- | --- |
|  |  |  |  |
|  |  |  |  |

If you have found antiSMASH useful, please cite us.
